# Functional characterization and analysis of transcriptional regulation of sugar transporter *SWEET13c* in sugarcane *Saccharum spontaneum*

**DOI:** 10.1186/s12870-022-03749-9

**Published:** 2022-07-22

**Authors:** Xiuting Hua, Qiaochu Shen, Yihan Li, Dong Zhou, Zhe Zhang, Sehrish Akbar, Zhengchao Wang, Jisen Zhang

**Affiliations:** 1 State Key Laboratory for Conservation and Utilization of Subtropical Agro-Bioresources & Guangxi Key Laboratory of Sugarcane Biology, Guangxi University, Nanning, 530004 China; 2 Center for Genomics and Biotechnology, Haixia Institute of Science and Technology, Fujian Provincial Key Laboratory of Haixia Applied Plant Systems Biology, College of Life Sciences, National Engineering Research Center for Sugarcane, Fujian Agriculture and Forestry University, Fuzhou, 350002 China; 3 Key Laboratory of Sugarcane Biology and Genetic Breeding, Ministry of Agriculture, Fujian Agriculture and Forestry University, Fuzhou, 350002 China; 4 College of Life Sciences, Fujian Normal University, Fuzhou, 350117 China

**Keywords:** *Saccharum spontaneum*, Sugar transport, *SWEET13c*, Transcription factor

## Abstract

**Background:**

Sugarcane is an important crop for sugar production worldwide. The Sugars Will Eventually be Exported Transporters (SWEETs) are a group of sugar transporters recently identified in sugarcane. In *Saccharum spontaneum*, SsSWEET13c played a role in the sucrose transportation from the source to the sink tissues, which was found to be mainly active in the mature leaf. However, the function and regulation of SWEETs in sugarcane remain elusive despite extensive studies performed on sugar metabolism.

**Results:**

In this study, we showed that *SsSWEET13c* is a member of SWEET gene family in *S. spontaneum*, constituting highest circadian rhythm-dependent expression. It is a functional gene that facilitates plant root elongation and increase fresh weight of *Arabidopsis thaliana*, when overexpressed. Furthermore, yeast one-hybrid assays indicate that 20 potential transcription factors (TFs) could bind to the *SsSWEET13c* promoter in *S. spontaneum*. We combined transcriptome data from developmental gradient leaf with distinct times during circadian cycles and stems/leaves at different growth stages. We have uncovered that 14 out of 20 TFs exhibited positive/negative gene expression patterns relative to *SsSWEET13c*. In the source tissues, *SsSWEET13c* was mainly positively regulated by *SsbHLH34*, *SsTFIIIA-a*, *SsMYR2*, *SsRAP2.4* and *SsbHLH035*, while negatively regulated by *SsABS5*, *SsTFIIIA-b* and *SsERF4*. During the circadian rhythm, it was noticed that *SsSWEET13c* was more active in the morning than in the afternoon. It was likely due to the high level of sugar accumulation at night, which was negatively regulated by *SsbZIP44*, and positively regulated by *SsbHLH34*. Furthermore, in the sink tissues, *SsSWEET13c* was also active for sugar accumulation, which was positively regulated by *SsbZIP44*, *SsTFIIIA-b*, *SsbHLH34* and *SsTFIIIA-a*, and negatively regulated by *SsERF4*, *SsHB36*, *SsDEL1* and *SsABS5*. Our results were further supported by one-to-one yeast hybridization assay which verified that 12 potential TFs could bind to the promoter of *SsSWEET13c*.

**Conclusions:**

A module of the regulatory network was proposed for the *SsSWEET13c* in the developmental gradient of leaf and circadian rhythm in *S. spontaneum*. These results provide a novel understanding of the function and regulation of *SWEET13c* during the sugar transport and biomass production in *S. spontaneum*.

**Supplementary Information:**

The online version contains supplementary material available at 10.1186/s12870-022-03749-9.

## Background

High crop yields are beneficial for food production, yet the underlying mechanism that determine crop yields is still not fully understood. Through absorbing light energy, plants leaf can fix carbon to form photosynthetic products (carbohydrates) [1]. Sugars, is one of the primary carbohydrate form [2]. In plants, 35% ~ 40% of sugars are consumed to provide energy for various growth activities including cell elongation, division, differentiation, nutrient absorption, and plant development. Majority of sugars function as metabolic intermediates in cells (monosaccharides, amino acids, organic acids, etc.) and the remaining sugars are stored in the vacuole as the original formation, fixed to a mobile polymer (plasmid starch), or added to structural biomass (cellulose, hemicellulose and lignin) [3].

Sucrose is synthesized in mesophyll cells and diffuses from cell to cell via plasmodesmata or a long-distance transportation. Hereafter sucrose could be transferred from the phloem parenchyma cells to the apoplast and loaded into the phloem sieve element/companion cells (SEs/CCs) via sucrose transporters [4–8]. Sugars Will Eventually be Exported Transporter (SWEETs)/sucrose transporters (SUTs) are a series of crucial transporters that play essential roles in sucrose transport pathways [9, 10]. SWEET proteins with seven transmembrane helices are identified as a novel group of sugar transporters that facilitate the transport of sugars like glucose, fructose and/or sucrose bi-directionally. SWEETs participate in numerous metabolic and physiological processes in plants, including phloem loading, nectar secretion, pollen nutrition, bacterial infection, senescence, seed filling and copper transport [11]. In *Arabidopsis thaliana*, researchers have identified 17 members of *SWEET* gene family [12]. *AtSWEET9* expresses at the nectary and possesses a negative correlation with the starch content in plants, which is required for nectar secretion [13, 14]. *AtSWEET13* and *AtSWEET14* mediate cellular gibberellin uptake to regulate anther dehiscence, as well as seeds and seedling phenotypes [15]. *AtSWEET11* and *AtSWEET12*, presenting on the plasma membrane of phloem parenchyma cells, mediate phloem loading of sucrose [16]. As the closest homolog gene of *AtSWEET11* and *AtSWEET12*, *AtSWEET13* has higher expression in *Atsweet11*;*12* double mutant plants. Moreover, *Atsweet11*;*12* double mutants have lower levels of starch accumulation in leaves and radioactive tracer efflux from the petioles, mirroring *SWEET11* and *SWEET12* cooperatively transport sucrose out of leaves [16]. In rice (*Oryza sativa* L.), the *SWEET* gene family consists of 21 members [17]. *OsSWEET11* can not only transport sucrose but contributes to grain filling. *OsSWEET13* functions as the targeted susceptible gene for *PthXo2* of *X. oryzae pv. oryzae* in the bacterial blight disease. Additionally, *OsSWEET11*, *12* and *14* are verified as the targets of *Xoo* effectors [18, 19]. *Osdof11* (DNA binding with one finger) mutation exerts a stunted phenotype due to reduced sucrose transported by low expression of both *SUT* and *SWEET* genes [20]. In maize (*Zea mays* L.), *ZmSWEET13s* paralog (a, b, c) triple knock-out mutants exhibit severely reduced photosynthesis abilities and dysregulated glucose metabolism [21].

Sugarcane (*Saccharum* spp., Poaceae), a perennial and tropical/subtropical crop, contributes to approximately 80% sugar and 40% of global ethanol production for humans [22]. As a typical C4 crop, sugarcane displays high efficiency on the utilization of water, nitrogen, and light compared to C3 crops, leading to greater yields when confronted with intense light, low carbon dioxide concentration and drought conditions. The sugarcane’s hyperploid and large genome have slowed down the research within sugar-related fields.

Recently, we identified 22 members of *SWEET* gene family in *S. spontaneum* [23], among which *SWEET13s* (a, b, c) were found to be duplicated genes. Noticeably, *SWEET13c* is increased by 1001-fold expression from the leaf base to tip, which was consistent with photosynthesis intensity (from weak to strong) through the blade. During the diurnal cycle, *SWEET13c* had maximal and minimum expression at 6:00 and 18:00, respectively. Additionally, 2.15-fold change of *SWEET13c* transcript abundance was detected,,suggesting that it is involved in diurnal rhythms and functions as a strong candidate gene which is supporting sucrose transport from source to sink in *S. spontaneum* [23]. Here, we focus on the *SsSWEET13c* to explore its ability to transport sugars and facilitate plant growth. We also aimed to identify the potential transcription factors (TFs) binding to the upstream of *SsSWEET13c*. Furthermore, we assessed the expression pattern of all identified TFs. These findings could help us further reveal the physiological effects and regulatory mechanisms of *SWEET13c* in *S. spontaneum*.

## Results

### *SsSWEET13c* overexpression reduces contents of sugars in leaves

Given that the transformation system of sugarcane is still unsolved, to examine whether SsSWEET13c can transport sugars, we conducted experiments in a transgenic *A. thaliana* over-expressing *SsSWEET13c* driven by the constitutive *Cauliflower mosaic virus* (CaMV) 35S promoter. Transgenic lines were screened to the homozygous T3 generation. Results of RT-qPCR showed that six lines of *35S*_*pro*_: *SsSWEET13c* presented higher expression levels relative to the *A. thaliana Columbia ecotype* (wild-type, WT) (Additional file 1). Considering the variation of overexpression folds, the average expressional value of these six *35S*_*pro*_: *SsSWEET13c* lines was utilized through consecutive measures (Additional file 1).

**Fig. 1:**
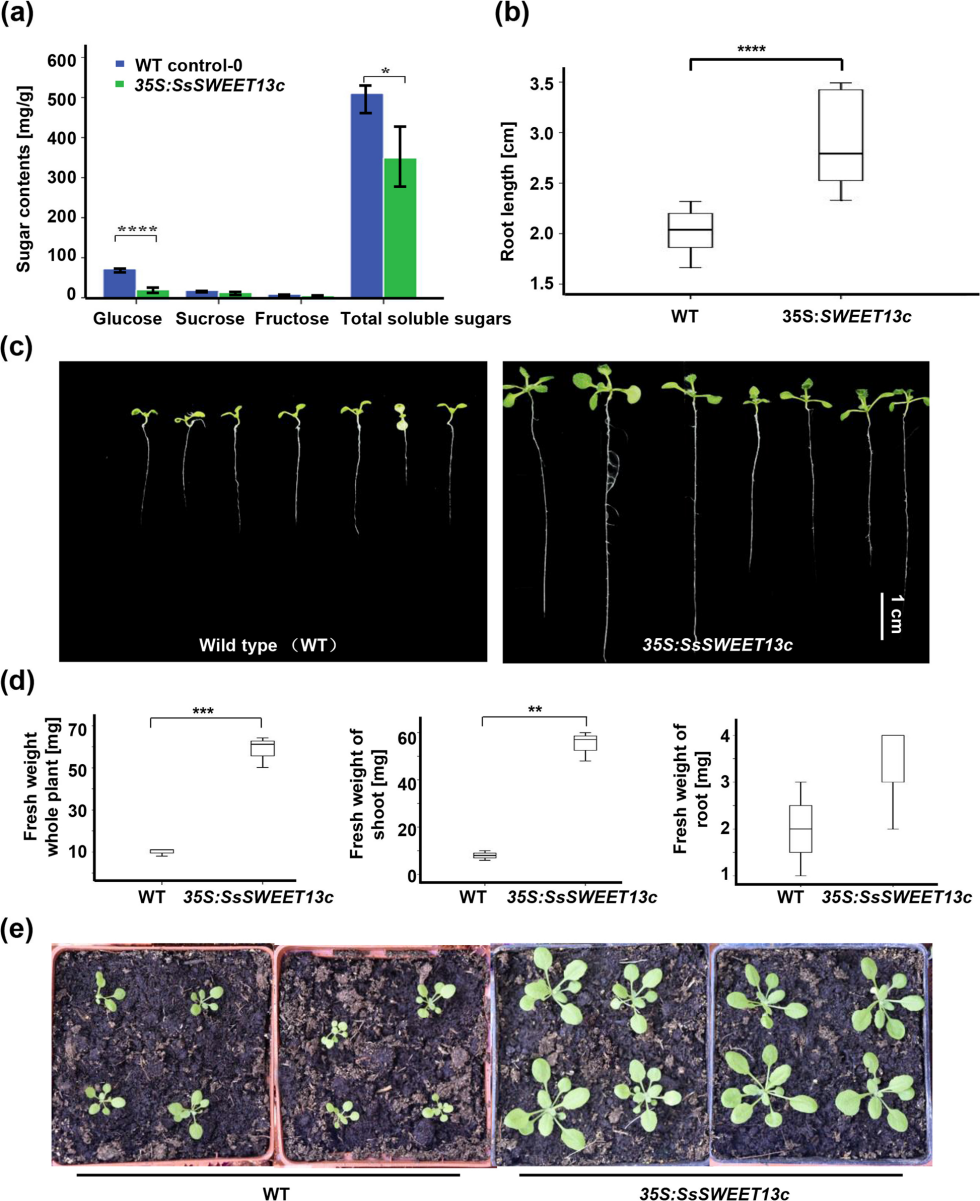
The measurement of sugar contents, root length and fresh weight for wild-type and *35S:SsSWEET13c* of *Arabidopsis* plants. **a** sugar contents **b** root length (7-days old). **c** root phenotype (7-days old). **d** fresh weight (15-days old). **e** plant phenotype (15-days old). *, *p* < 0.05. **, *p* < 0.01. ***, *p* < 0.001. ****, *p* < 0.0001

The leaves of WT and overexpression plants were collected to allow the determination of the contents of glucose, sucrose, fructose and total soluble sugar after 21 days of growth. The results suggested that *SsSWEET13c* overexpression plants possessed a significant alteration in the sugar-transporting ability relative to WT. For glucose content, the WT plants contained 76.3 mg g^−1^, while the *SsSWEET13c* overexpression plants contained 19.3 mg g^−1^, which was significantly reduced (*p* < 0.0001) (Fig. 1a). Compared with 18.0 mg g^−1^ of the WT plants, the average sucrose level of *SsSWEET13c* overexpression plants was 13.1 mg g^−1^ (Fig. 1a). Regarding the fructose content, the average level of 5.2 mg g^−1^ in *SsSWEET13c* overexpression plants was lower than that of 7.2 mg g^−1^ in the WT (Fig. 1a). Regarding the total soluble sugar contents, WT plants contained 510.2 mg g^−1^, whereas a significant reduction to 348.1 mg g^−1^ was observed in *SsSWEET13c* overexpression plants (*p* < 0.05) (Fig. 1a). These results indicated that *SsSWEET13c* could assist in transporting the produced sugar,lead to reduction of sugar concentration.

### *SsSWEET13c* overexpression enhances the root length and fresh weight

For analysis of sugar content, we took eight seeds from WT and six over-expression *SsSWEET13c* lines and observed for 7 d for any changes in root elongation. The results suggested that the average root length of *SsSWEET13c* overexpression lines was 3.41 cm, with the longest root being 5.54 cm which is significantly longer than the 2.22 cm of the WT (Fig. 1b, c and Additional file 2). Furthermore, we transferred the *A. thaliana* plants into nutrient soil to cultivate for additional 7 d (at 23 °C and 16 h/8 h light/dark), We measured average fresh weight of these WT and *SsSWEET13c* overexpression lines including the whole plant weight, root weight and shoot weight. In the Arabidopsis WT, the average fresh weight of the whole plants, shoots and roots were 9.53 mg, 7.73 mg and 1.90 mg, respectively (Fig. 1d and Additional file 2). However, in *SsSWEET13c* overexpression lines, the average fresh weights were 58.0 mg (whole plants), 54.7 mg (shoots) and 3.2 mg (roots) (Fig. 1d, e). These results demonstrated that *SsSWEET13c* is responsible for increase increased in root length and fresh weight (Fig. 1b-e).

Next, we correlated the differential accumulation of sugar (glucose, sucrose, fructose and total soluble sugar) in the leaves with biomass accumulation (length of root and weight of root, shoot and whole plant). We found out that, in comparison with WT, sugar content in leaves of overexpression *SsSWEET13c* plants decreased by more than 1.38-fold change, while biomass accumulation increased by more than 1.65-fold change (Table 1). This indicated that sugar content in leaves were negativey correlated with biomass accumulation.

**Table 1:** The biomass and the sugar in the leaves

	***35S***_***pro***_**: *SsSWEET13c***	**wild-type**	**fold change**	**1/(fold change)**
**glucose (mg g-1)**	19.30	76.30	0.25	3.95
**sucrose (mg g-1)**	13.10	18.00	0.73	1.37
**fructose (mg g-1)**	5.20	7.20	0.72	1.38
**soluble sugar (mg g-1)**	348.10	510.20	0.68	1.47
**root length (cm)**	3.37	2.04	1.65	0.61
**root (mg)**	3.30	1.90	1.74	0.58
**shoot (mg)**	54.60	7.90	6.91	0.14
**whole plant (mg)**	57.90	9.80	5.91	0.17

### Yeast one-hybrid identifies 20 TFs regulating *SWEET13c*

Although the ability of *SsSWEET13c* to transport sugars and enhanced plant growth had been preliminarily determined. Further, we interested in the TFs which are regulating *SsSWEET13c* and their gradual increase in expression level from the leaf base to tip, and a decrease in expression during the day followed by rising expression level at night. The prediction of TF binding sites was initially performed by the PlantCare database. In total, we predicted 27 potential TF binding sites in a 2 kb region upstream from the promotor of *SsSWEET13c* (Table 2). Among them, 15 TFs binding sites, including three Box 4, two chs-CMA1a, four G-Box, TCCC-motif, GT1-motif, MRE, TCT-motif, ATC-motif, and 3-AF1, are predicted to be responsive to light. Three binding sites, CGTCA-motif, TGACG-motif and ABRE, were predicted as hormone-responsiveness elements, and two MBS binding sites were predicted as drought-inducibility elements (Table 2).

**Table 2:** Prediction of *cis* elements for the 2000 bp promoter region of *SsSWEET13c*

Site Name	Function	*SsSWEET13c*-Promoter	Site
3-AF1 binding site	light responsive element	TAAGAGAGGAA	-154
ABRE	abscisic acid responsiveness	ACGTG	-1355
ABRE	abscisic acid responsiveness	ACGTG	-1476
ABRE	abscisic acid responsiveness	CACGTG	-1477
ARE	anaerobic induction	AAACCA	-1418
ARE	anaerobic induction	AAACCA	-1899
ARE	anaerobic induction	AAACCA	-1943
ATC-motif	light responsiveness	AGCTATCCA	-395
Box 4	light responsiveness	ATTAAT	-339
Box 4	light responsiveness	ATTAAT	-463
Box 4	light responsiveness	ATTAAT	-1510
CAT-box	meristem expression	GCCACT	-983
CCAAT-box	MYBHv1 binding site	CAACGG	-539
CGTCA-motif	MeJA-responsiveness	CGTCA	-1810
chs-CMA1a	light responsive element	TTACTTAA	-1492
chs-CMA1a	light responsive element	TTACTTAA	-1517
G-box	light responsiveness	CACGAC	-6
G-box	light responsiveness	CACGTC	-1356
G-box	light responsiveness	CACGTG	-1477
G-Box	light responsiveness	CACGTG	-1477
GCN4_motif	endosperm expression	TGAGTCA	-1277
GT1-motif	light responsive element	GGTTAA	-817
MBS	drought-inducibility	CAACTG	-74
MBS	drought-inducibility	CAACTG	-86
MRE	light responsiveness	AACCTAA	-741
TCCC-motif	light responsive element	TCTCCCT	-184
TCT-motif	light responsive element	TCTTAC	-631
TGACG-motif	MeJA-responsiveness	TGACG	-1810

**Fig. 2:**
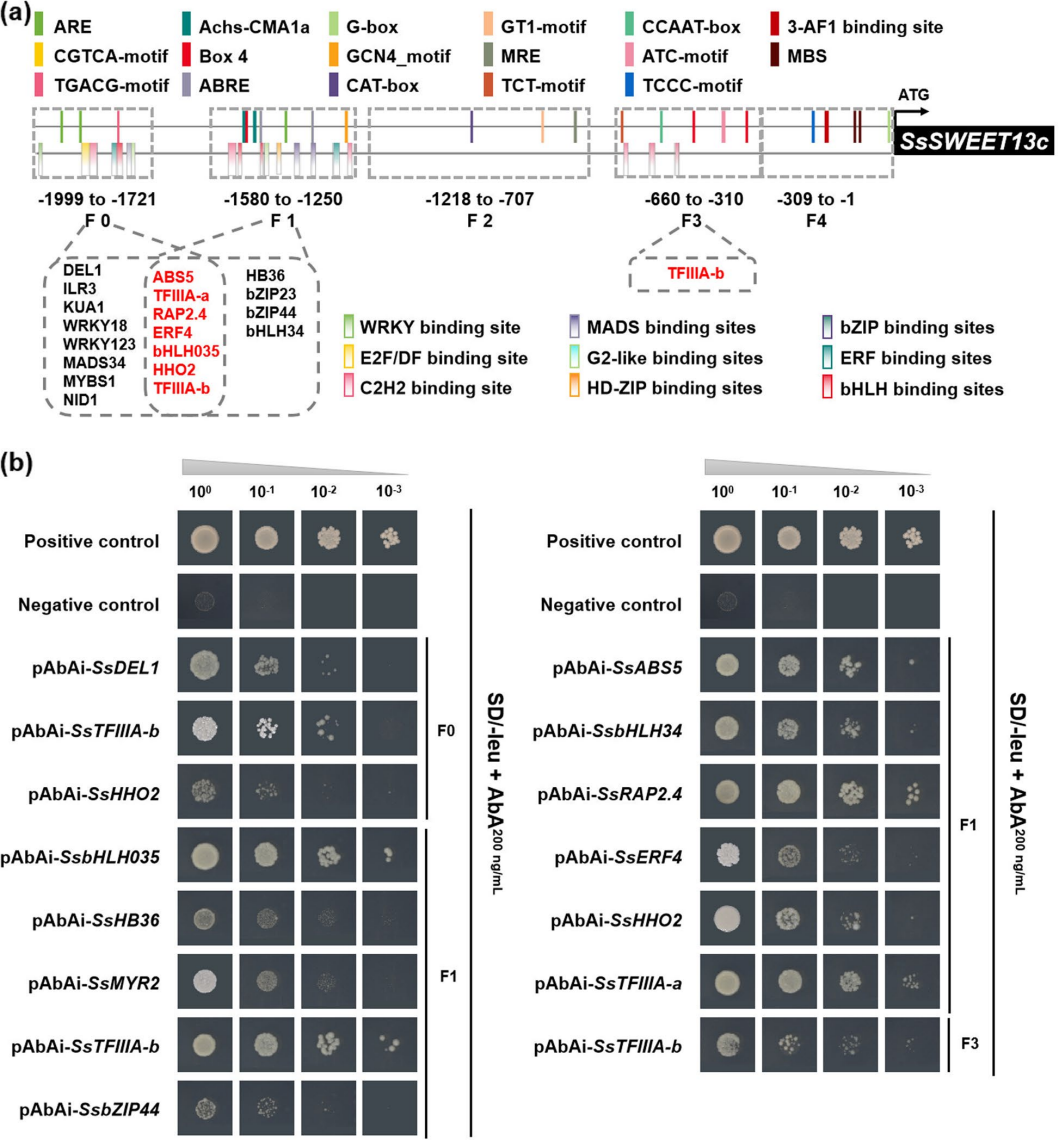
Analysis of the transcription factors (TFs) binding to the *SsSWEET13c* upstream region. **a** Schematic representation of the *SsSWEET13c* upstream region divided into five fragments (F0 to F4) that were used as baits in the yeast one-hybrid (Y1H) screening, and the TFs identified. Positions of the *SsSWEET13c* start codon (ATG) are indicated. **b** The One-to-One Yeast hybrid results for nine TFs binding to promoter of *SsSWEET13c*. 100: original yeast solution, 10^–1^: diluted by 10 times, 10^–2^: diluted by 100 times, 10^–3^: diluted by 1000 times

To further determine TFs binding role at the promoter of *SsSWEET13c*, a yeast one-hybrid (Y1H) experiment was performed. To begin, the promoter sequence was divided into five short fragments, named F0 (-1999 to -1721), F1 (-1580 to -1250), F2 (-1218 to -707), F3 (-660 to -310) and F4 (-309 to -1) (Fig. 2, Additional file 3). Except for F2, the remaining four segments containing the core motifs in promoters of the target gene were cloned upstream of the Aureobasidin (AbA) resistance gene (Additional file 4) and transformed intoY1H Gold strains as a bait. As a result, the self-activation of three bait fragments was successfully inhibited. The inhibition concentration was 200 ng/µL for F0, F1 and F3. Next, yeast competent cells with the promoter fragments of *SsSWEET13c* were transformed with *S. spontaneum* cDNA library plasmids, as well as the positive (pAbAi-53) and negative controls, which were cultivated on the basic SD medium lacking the Leu with a corresponding AbA level. Yeast cells grown were screened for a second time under the same cultivated conditions. Finally, using the universal primers of the vector pGADT7 for DNA sequence, we obtained sequencing results of segments interacting with promoters of *SsSWEET13c* and 97 gene annotation results based on the BLAST search (https://www.ncbi.nlm.nih.gov/). Of those, 20 genes were summarized as potential TFs regulating *SsSWEET13c *via aligning using PlantRegMap/PlantTFDB v5.0 [24] (Additional file 5 and Additional file 6), including *SsMYR2*, *SsMADS34*, *SsWRKY18*, *SsHB36*, *SsABS5*, *SsbZIP44*, *SsHHO2*, *SsMYBS1*, *SsNID1*, *SsRAP2*.*4*, *SsbHLH035*, *SsDEL1*, *SsbHLH34*, *SsERF4, SsILR3*, *SsKUA1*, *SsTFIIIA*-a, *SsTFIIIA*-b, *SsWRKY123* and *SsbZIP23.*

### Expressional patterns of the TFs regulating *SsSWEET13c*

To test the expression patterns of TFs summarized from the Y1H experiment on *SsSWEET13c*, we conducted RNA-seq analyses in *S. spontaneum* at three RNA-seq datasets: 1) different developmental stages, 2) leaf developmental gradient and 3) diurnal cycles (Additional file 7).

### Expression pattern of TFs at different developmental stages

**Fig. 3:**
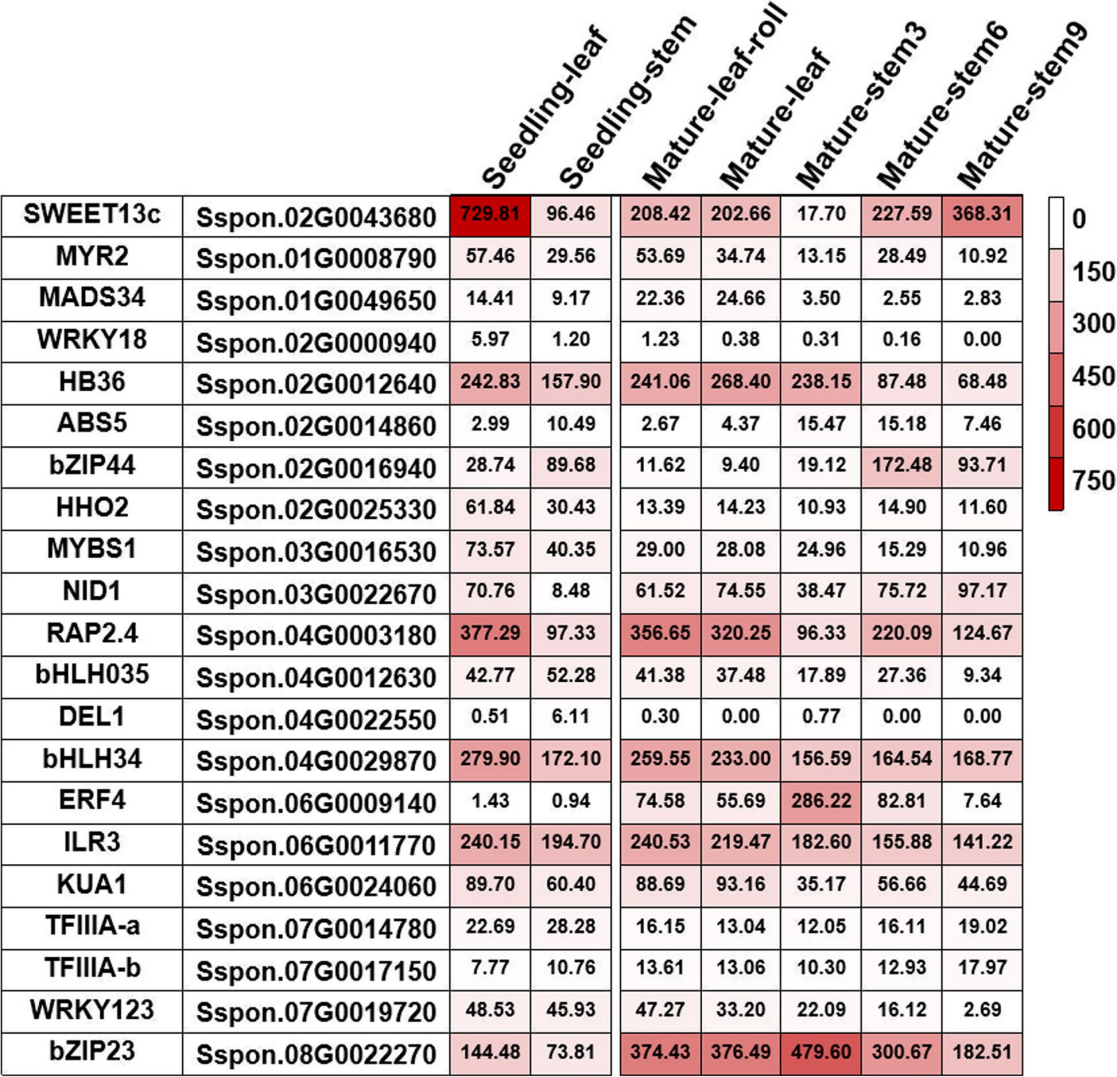
The expression profile for potential transcription factors (TFs) regulating *SsSWEET13c* based on Transcript Per Million (TPM) in leaf and stem at seedling stage and mature stage in *Saccharum spontaneum*

To explore the regulation of TFs on *SsSWEET13c* at different developmental stages, we performed RNA-seq at the seedling and mature stages, including the seedling leaf, seedling stem, mature leaf, and mature stems (from top to bottom: stem 3, stem 6 and stem 9) (Fig. 3). *SsSWEET13c* displayed 3.6-fold enhanced expression in the seedling leaf compared with that in the lowest tissue of mature leaf,. Expressional levels in the stem 3, 6 and 9 gradually increased by 20.81-fold change expression, which implies *SsSWEET13c* could be associated with leaf development and secondary cell wall cellulose synthesis. Among these TFs, *SsHB36* was mainly expressed in the leaves which was 3.92-fold higher than that in the stems, *SsERF4* was mainly expressed in stem 3 of mature stages which is 3.84-fold higher than its expression in the leaves and 3.46-fold higher than that in the stem 9. *SsbZIP44* was mainly expressed in the stem. and it increased 3.12-fold and 4.90-fold expression in seedling stem and mature stem compared with that in seedling leaves and mature leaves individually (Fig. 3). *SsRAP2.4*, *SsbHLH34*, *SsILR3* and *SsbZIP23* exhibited a high expression level in both seedling and mature leaves.

### Expression pattern of TFs at leaf developmental gradient

**Fig. 4:**
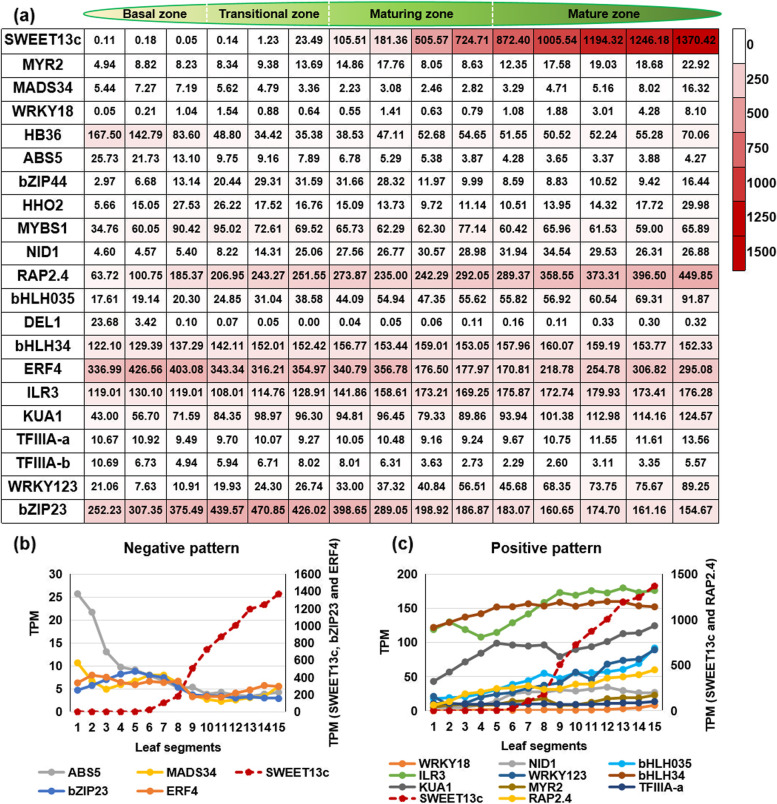
The expression profile and patterns for potential transcription factors (TFs) regulating *SsSWEET13c* based on TPM across leaf developmental gradients in *Saccharum spontaneum*. **a** expression profile **b** transcription factors (TFs) with negative expression patterns relative to those in *SsSWEET13c* **c** TFs with positive expression patterns relative to those in *SsSWEET13c*

To investigate the regulation of TFs on *SsSWEET13c* expressions in the leaves of source tissues, the transcriptome analyses and expression profiles were performed during the leaf developmental gradient (Fig. 4a). Corresponding to the maize leaf developmental gradient [25], we divided the second leaf at 11-day old with 15 cm length of *S. spontaneum* into 15 identical segments (each segment of 1 cm). Simultaneously, the *S. spontaneum* leaf was parted into four zones, namely a basal zone (base, the sink tissue), a transitional zone (6 cm, the sink-source transition), a maturing zone (10 cm), and a mature zone (tip, active C4 photosynthesis). Four out of 20 TFs, including *SsABS5*, *SsERF4*, *SsTFIIIA-b* and *SsbZIP23* (Pearson Correlation, PC ≥|0.5|, *p* < 0.05) displayed negative expression patterns relative to those in *SsSWEET13c* across the leaf developmental gradient, and the gene expressional levels gradually decreased from the leaf base with low photosynthesis to the leaf tip with active photosynthesis (Fig. 4b, Additional file 8). Nine out of 26 TFs, including *SsMYR2*, *SsWRKY18*, *SsNID1*, *SsRAP2.4*, *SsbHLH035*, *SsbHLH34*, *SsILR3*, *SsKUA1*, *SsTFIIIA-a* and *SsWRKY123* (PC ≥|0.5|, *p* < 0.05), displayed positive expression patterns relative to those in *SsSWEET13c* and the gene expression levels gradually increased from the foliar base to the tip (Fig. 4c). Expression patterns of *SsMYR2*, *SsHB36*, *SsbZIP44*, *SsDEL1*, *SsMYBS1*, *SsNID1*, *SsRAP2*.*4*, *SsbHLH34* and *SsKUA1* were verified by reverse transcription quantitative PCR (RT-qPCR) in three leaf segments from leaf gradients (Additional file 9).

### Expression pattern of TFs at diurnal cycles

**Fig. 5:**
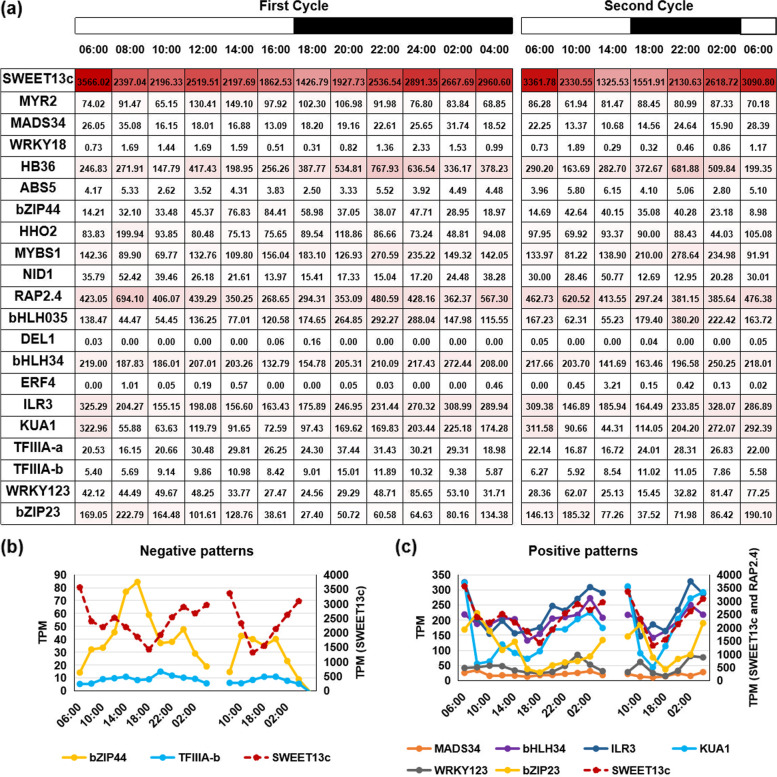
The expression profile and patterns for potential transcription factors (TFs) regulating *SsSWEET13c* based on TPM at different time points in *Saccharum spontaneum*. **a** expression profile **b** TFs with negative expression pattern relative to those in *SsSWEET13c* expression **c** TFs with positive expression pattern relative to those in *SsSWEET13c* expression

To further explore the regulatory patterns of TFs on *SsSWEET13c* during diurnal cycles, we conducted RNA-seq analyses in leaf samples every 2-h during the first 24-h and every 4-h during the second 24-h as an addition in *S. spontaneum* (Fig. 5a). Six out of 20 TFs, *SsMADS34*, *SsbHLH34*, *SsILR3*, *SsKUA1*, *SsWRKY123* and *SsbZIP23* (PC ≥|0.5|, *p* < 0.05), displayed positive expression patterns relative to those in *SsSWEET13c.* Their expressions gradually increased from 6:00 to 18:00 followed by a decrease from 18:00 to 04:00 h. (Fig. 5b); Two out of 20 TFs, including *SsbZIP44* and *SsTFIIIA-b* (PC ≥|0.5|, *p* < 0.05), displayed similar expression patterns to those in *SsSWEET13c*. The expression of these genes gradually declined from 06:00 to 18:00 h followed by an increase from 18:00 to 4:00 h. (Fig. 5c).

### One-to-one yeast hybridization verification of 12 potential TFs binding to the promoter of *SsSWEET13c*

To verify the most significant TFs determined by Y1H and transcriptome analysis, constructs containing the TFs fragments were extracted from the yeast strains screened by the Y1H secondary library and proliferated via* e.coli* competent propagation. Then,constructs were transformed into yeast competent cells with pBait-AbAi bait vector containing the corresponding promoter fragments and cultivated them for 2 ~ 3d. The yeast solution was serially diluted (10 times, 100 times and 1,000 times) for spotting, and p53-AbAi was used as a positive control. One-to-one verification results of Y1H successfully confirmed 12 TFs, named *SsDEL1*, *SsTFIIIA*-*b*, *SsHHO2*, *SsbHLH035*, *SsHB36*, *SsMYR2*, *SsbZIP44*, *SsABS5*, *SsbHLH34*, *SsRAP2*.4, *SsERF4* and *SsTFIIIA*-a. In addition, the *SsWEET13c *promoter was assessed for TF binding sites (TFBS) in F0, F1 and F3 to which TFs might bind (Fig. 2a). We have found 12 TFs with their predicted TFBS in the corresponding fragment, indicating that these 12 TFs could bind to the promoter and regulate the expression of *SsSWEET13c* (Fig. 2b).

## Discussion

Sugarcane possesses complex genetic backgrounds due to its high polyploidy level. This high polyploidy is problematic due to unavailability of transgenic seedlings with characterized phenotypes. Therefore, in this study, we over-expressed *SsSWEET13c* in the model plant *A. thaliana* to identify the sugar transportability of *SsSWEET13c*.

In *S. spontaneum*, *SsSWEET13c* dramatically increased from the leaf maturing to tip zones (Fig. 4b). A recent study has found that *S. spontaneum SsSWEET13c* homolog *ZmSWEET13s* played primary roles in sucrose transport in maize [21]and as a possible key player had been attributed to C4 photosynthesis [26]. Noticeably, *ZmSWEET13s* showed the highest expression in leaf tips [21]. We thus deduced that *SWEET13c* was mainly involved in photosynthesis for sugar transport. Sugars are produced in the leaves, and which are then transported to other organs and act as signals that play crucial roles in plant growth and development [27]. Relative to wild type, sugar content in leaves of overexpressed *SsSWEET13c A. thaliana* discernibly decreased, while the root length/weight, shoot weight and the fresh weight increased significantly (Fig. 1). This may have been due to the enhanced sugar efflux from the leaf driven by *SsSWEET13c*, and transported sugar to the root and shoot [16]. Our results indicated that *SsSWEET13c* increased the photosynthesis ability for the growth and development. There were similar conclusions in previous studies, where overexpression pear *PbSWEET4* caused sugar reduction and early senescence in leaves [28]. Moreover, overexpression of *A. thaliana AtSWEET16* exhibited increased growth efficiency [29]. Overexpression Grapevine *VvSWEET4* was consistent with *SsSWEET13c* in *A. thaliana*, displaying enhanced root growth but distinct from those observed in *SsSWEET13c* in *A. thaliana*, *e.g*., higher glucose and fructose contents and higher radiolabeled glucose passive uptake in Grapevine [30].

Some crop yield depends on the efficient allocation of sucrose from leaves to seeds [21]. But the information on plant SWEET proteins acting as sugar transporters in seed development is limited. Seed of sweet mutants in rice has been shown to accumulated more sucrose, glucose or fructose but less starch [31]. This is in the agreement of our findings where we found that the overexpression of *SsSWEET13c* resulted in reduced sugar content. It was observed that *SsSWEET13c* in SWEETs clade III [23], while *AtSWEET13* and *AtSWEET14* were in clade III. Noticeably, sweet13;14 double mutants increased seedling and seed size [15], which was distinct from those observed in the triple zmsweet13 knock out mutants in maize. Moreover, *OsSWEET11* and *OsSWEET15* in clade III are expressed preferentially in the caryopsis and played a key role in seed filling with sucrose efflux functions in rice [18, 32]. Similarly, *OsSWEET11* is a sugar transporter with a key role in seed development [31]. Their knockout mutants plant of *OsSWEET11* showed severely shrunken mature caryopses [18, 32, 33]. SWEET in the same clade may perform divergent functions in monocots and dicots. We hypothesized that overexpression of *SsSWEET13c* in *A. thaliana* might involve in increased seed size by sugar efflux and starch production. Thus, *SsSWEET13c* might be one of the important candidates for the high biomass of *S. spontaneum*.

TFs can bind to promoter regions of the target gene at conserved sites, in turn regulating different physiological activities in plants. *Cis*-element prediction revealed that 15 light-responsive sites existed in the upstream 2 kb promoter of *SsSWEET13c* (Table 2). The prediction could be supported by the evidence of transcriptome dynamics and the expression patterns of TFs for the *SsSWEET13c*. As the expression of *SsSWEET13c* increased from the leaf base (fewer lights) to the tip (more lights), the conjecture is that the leaf segments with sufficient light create abundant glycogen through photosynthesis. Thus, a higher level of sugar transporter SsSWEET13c is essential for assisting the sugar transfer in *S. spontaneum* leaves. During circadian rhythm, the expression level of *SsSWEET13c* shows a gradual decrease and increase, This led us to speculate that SsSWEET13c will transport the carbohydrates, produced at the day-time, to the other tissues and organs at night to provide the necessary glycogen for the growth and respiration in *S. spontaneum*. In addition, 20 TFs were identified as potential regulators for the *SsSWEET13c* based on the Y1H, and at least eight of them are in response to circadian rhythm.

The verification of the Y1H self-activation experiment provided the optimal AbA concentration for the two screenings of the subsequent Y1H experiments and avoid false positives in the present experiment (Fig. 2b), Additionally 20 TFs were verified to be regulators for *SsSWEET13c.* Based on the expression patterns in the leaf segments (Fig. 4), four TFs show a negative regulation pattern, whereas ten TFs show a positive regulation pattern for *SsSWEET13c* expression. The negative TFs for the *SsSWEET13c* have a much lower expression level than the positive TFs (Fig. 4). Therefore, we suggested that the positive TFs are the main regulators regulating *SsSWEET13c*.

Based on a series of transcriptome analyses for potential TFs binding to the promoter of *SsSWEET13c*, 12 TFs (*SsABS5*, *SsbHLH035*, *SsbHLH34*, *SsbZIP44*, *SsDEL1*, *SsERF4*, *SsHB36*, *SsHHO2*, *SsMYR2*, *SsRAP2.4*, *SsTFIIIA-a* and *SsTFIIIA-b*) were further identified by one-to-one Y1H. The *SsbHLH035* is the closest ortholog to the *AT5G57150* in *A. thaliana*. The *AT5G57150* is expressed in roots and shoot, and showed strong differences in expression levels between the shoot and root (root/shoot > 20-fold) [34]. In this study, overexpression of *SsSWEET13c* increased the root length in *A. thaliana*. In *S. spontaneum*, the expression pattern of *SsbHLH035* along with the leaf gradient was similar to that of *SsSWEET13c* (PC = 0.89, *p* < 0.0001)(Fig. 3). Therefore, the regulation of *SsbHLH035* for *SsSWEET13c* may cause an increase in the root length. But further experiments are necessary for verification, such as detecting the expression of *SsbHLH035* in the roots, and further confirming the effect of *SsbHLH035* on *SsSWEET13c* for growth and development.

In *S. spontaneum*, the expression level of *SsbZIP44* peaked at 16:00 h, and the diurnal expression pattern was negative to that of *SsSWEET13c* (PC = -0.61, *p* = 0.0059). The close homologous gene of *SsbZIP44* is *AtbZIP44* in *A. thaliana*. *AtbZIP44* belonged to the S1 subgroup, which is translationally repressed by sucrose signaling [35]. Therefore, we assumed that *SsbZIP44* in *S. spontaneum* responded to sucrose signals and displayed negative regulation patterns to *SsSWEET13c*.

In *S. spontaneum*, the expression level of *SsRAP2.4* gradually increased from the leaf base to the tip and maximum at 8:00 h then decline at 16:00 h, It was found that expression level in the leaves being 1.62–3.70-fold higher than that in the stems (Figs. 3, 4, and 5). *SsRAP2.4* is a close homolog to *RAP2.4* in *A. thaliana*, which belongs to the DREB (dehydration-responsive element-binding proteins) subfamily of AP2/ERF proteins [36]. *RAP2.4* is involved in senescence control, as well as ethylene and cytokinin-related developmental processes [37]. Overexpression of *RAP2.4* results in root hair formation, hypocotyl and root elongation [37]. *SsSWEET13c* may also play a function associated with cytokinesis as the overexpression of the *SsSWEET13c* in plant increase the root length and the fresh weight. Therefore, *SsRAP2.4* was assumed to play a role in promoting cytokinesis by regulating *SsSWEET13c* in the *S. spontaneum* in different tissues.

*SsHB36* TF is the closest ortholog of *A. thaliana ATHB16*, belonging to the HD-ZIP TF family. It is reported that *ATHB16* mRNA was detected in all organs examined and acted as a growth regulator. However, reduced levels of *ATHB16* expression in transgenic Arabidopsis plants caused an increase in leaf cell expansion [38]. In *S. spontaneum*, *SsHB36* was highly expressed in the leaf base which was 2.40-fold higher than its expression in the leaf tip, and the expression level in the stem 3 was 3.48-fold higher than that in the stem 9. Leaf base and stem 3 are active parts for the growth and development in *S. spontaneum* (Figs. 3– 4). Therefore, *SsHB36* might be able to participate in shoot growth by regulating *SsSWEET13c*, and providing the necessary energy (sucrose) for cell elongation.

**Fig. 6:**
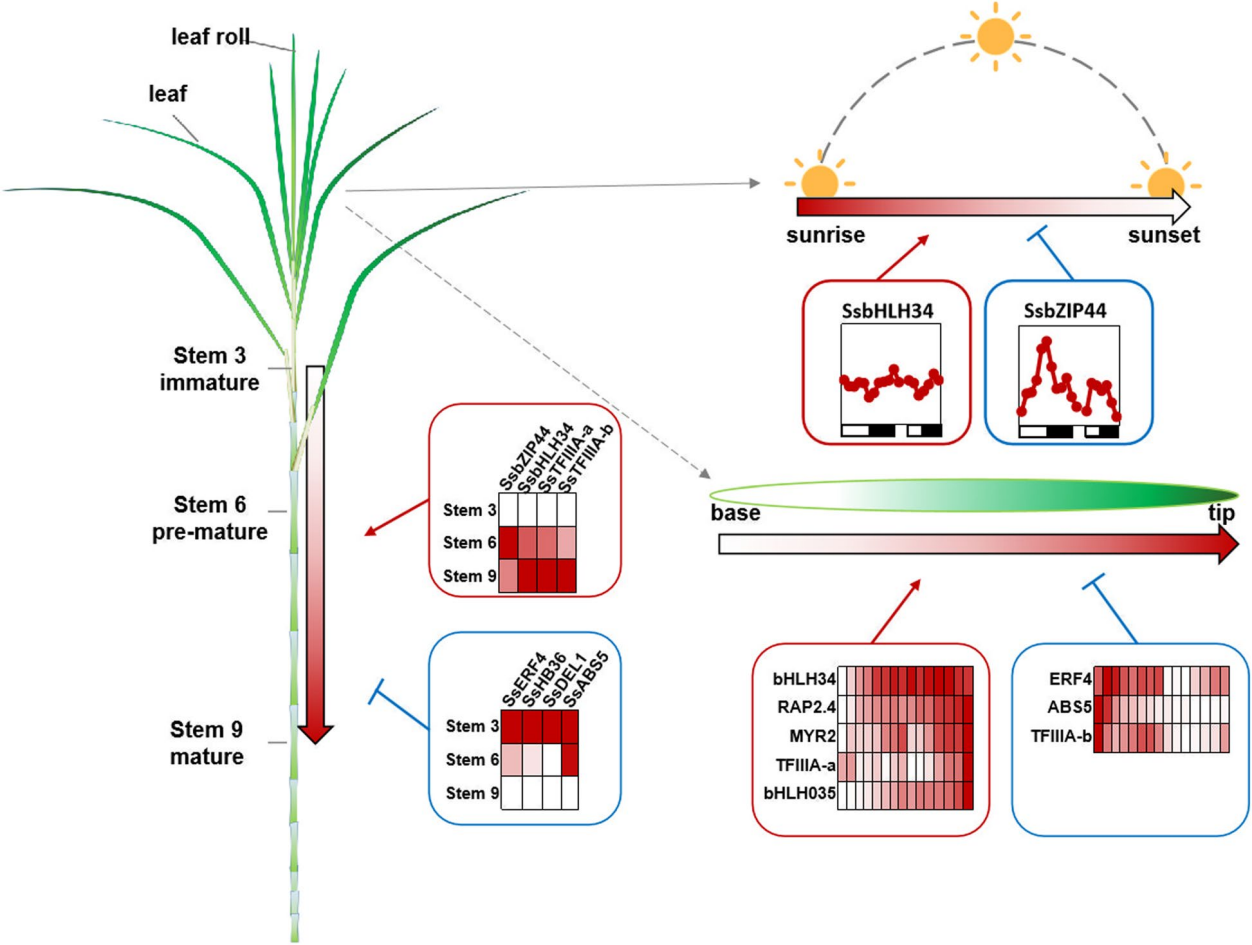
The proposed model of expression and regulation of transcription factors (TFs) binding to the promoter of *SsSWEET13c* in developmental leaves, circadian rhythm, and different stems of *Saccharum spontaneum*

Overall, with regards to the function for *SsSWEET13c* and its potential TFs, based on the gene expression profiles we proposed a regulatory network for the *SsSWEET13c* (Fig. 6). In *S. spontaneum*, *SsSWEET13c* played a role in the sucrose transportation from the source to the sink tissues and was mainly active in the mature leaf. In the source tissues, *SsSWEET13c* was mainly positively regulated by *SsbHLH34*, *SsTFIIIA-a*, *SsMYR2*, *SsRAP2.4* and *SsbHLH035*, while negatively regulated by *SsABS5*, *SsTFIIIA-b* and *SsERF4*. During the circadian rhythm, *SsSWEET13c* was more active in the sunset which was likely due to the high level of sugar accumulation at night. While, *SsSWEET13c* was negatively regulated by *SsbZIP44*, and positively regulated by *SsbHLH34*. In the sink tissues, *SsSWEET13c* was also active for sugar accumulation, which was positively regulated by *SsbZIP44*, *SsTFIIIA-b*, *SsbHLH34* and *SsTFIIIA-a*, and negatively regulated by *SsERF4*, *SsHB36*, *SsDEL1* and *SsABS5*. Nevertheless, this speculation still needs to be verified by tissue-specific expression and localization of these genes. Furthermore, interactions between the TFs and *SsSWEET13c* promoter could be further verified by other approaches as well such as electrophoretic mobility shift assay (EMSA).

## Conclusions

In conclusion, our study demonstrated that excessive *SsSWEET13c* will decrease sugar contents in leaves and boost the root length and the fresh weight at the same time in plants. Additionally, 20 TFs with the ability to bind to the *SsSWEET13c* promoter were identified through Y1H. Furthermore, our transcriptome analyses of TFs on developmental gradient leaf, distinct times during circadian cycles and stems/leaves at different growth stages generate potential expression patterns and regulatory network between these TFs and *SsSWEET13c*. Together, these results help us to explain the underlying processes of *SsSWEET13c* and its impact on breeding and the generation of high-quality sugar and biomass.

## Materials and methods

### Plant material and treatment

The founding *Saccharum* species, *S. spontaneum* (SES 208, 2n = 8x = 64, originated in the USA) were utilized in this study. The plant material was identified by Irvine JE [39]. The plants were cultivated in the greenhouse (14 h light 30 °C/10 h darkness 22 °C, 60% relative humidity), at the Center for Genomics and Biotechnology, Fujian Agriculture and Forestry University (FAFU, Fuzhou, China). The mature plants were planted in a field on the FAFU campus [40]. The collection of *S. spontaneum* and the performance of experimental research on such plant were complied with the national guidelines of China. We declared that *S. spontaneum* used for this study do not require application for special permissions.

The expression analyses for *S. spontaneum*, including developmental leaf segments, different times, leaves and stems at different periods, were performed as described in previous studies [23, 41, 42].

Seeds of *A. thaliana* Columbia ecotype were stored in an incubator (26 °C, 80% relative humidity) in the Center for Genomics and Biotechnology (FAFU, Fuzhou, China); transgenic lines *35Spro:SsSWEET13c* were acquired by two consecutive screenings. All the seedlings were incubated on the MS medium at 4 °C for one day, then transferred to the greenhouse (24 °C, 16 h/8 h light/dark, and 80% relative humidity) for seven days. The seedlings were then transferred to nutrient soil (PINDSTRUP 0-10 mm) for growth (24 °C, 16 h/8 h light/dark, and 80% relative humidity).

### RNA isolation, reverse transcription and cloning of *SsSWEET13c*

Total RNA was extracted using TRIzol reagent (Invitrogen, Shanghai, China). Acquired RNA quality was detected by the agarose gel electrophoresis and the NanoDrop 2000 spectrophotometer (keep OD260/OD280 = 1.8 ~ 2.0, OD260/OD230 ≈ 2.0). Good quality RNA was reverse-transcribed to first strand cDNA using PrimeScript™ 1st Strand cDNA Synthesis Kit (Takara, Dalian, China). The primers of *SsWEET13c* (Additional file 10) were designed via both Snap Gene Viewer and Primer Premier 5.0 and the PCR was performed with PrimeSTAR® Max DNA Polymerase (Takara, Dalian, China).

### Vector construction and plant transformation of *SsSWEET13c*

Using the Gateway method, the *SsSWEET13c* full-length cDNA was recombined into the pDONR207 vector by the Gateway entry clone. The pMDC140 vector was then used for the expression clone of *SsSWEET13c* and the CaMV35S promoter. Hygromycin B was the selectable marker employed. Constructs were transformed into *Agrobacterium tumefaciens* GV3101 and then introduced into WT Arabidopsis by the floral dip method [43]. At the T3 generation, homozygous transgenic lines were used for further assays.

### Quantitative reverse transcription PCR (RT-qPCR)

RNA extraction and the first-strand cDNA synthesis were performed in accordance with the above methods. Integrated DNA Technologies (http://www.idtdna.com/Primerquest/Home/Index) were used to design gene-specific primer pairs (Additional file 11). SYBR Green method was utilized for RT-qPCR. The product was purchased from the company of GenStar (A310-02). The 25S ribosomal RNA and glyceraldehyde-3-phosphate dehydrogenase (GAPDH) were taken as housekeeping genes. The reaction system as follows: 10µL of 2 × RealStar Green Fast Mixture, 0.5 µL of 50 ng/µL cDNA template, 0.5 µL of 10 mM forward primer, 0.5 µL of 10 mM reverse primer 0.5 µL, 8.5 µL of RNase-free H2O. BioRad CFX Connect Real-Time system was used with the following program: 2 min of denaturation at 95 ℃, followed by 40 cycles of 15 s at 95 ℃, 15 s at 60 ℃ and 30 s at 72 ℃. The relative expression levels for *SsSWEET13c* gene were calculated using the 2-^ΔΔCt^ method [44].

### DNA isolation, sequence analysis and molecular cloning of the promoter

Total genomic DNA was extracted by CTAB method. The upstream 2,000 bp promoter sequence of *SsSWEET13c* was selected for the *cis*-regulatory element online prediction via PlantCare [45]. The promoter sequence was divided into 5 shorter sequences to amplify them from the sugarcane genomic DNA.

### Measurement of sugar, root length and fresh weight content

Measurement kits for glucose, sucrose fructose and the total soluble sugar contents were purchased from Nanjing Jiancheng Bioengineering Research Institute (Nanjing, China). The glucose content was determined by the glucose oxidase method [46]. Glucose measurement kit includes standard solution, calibration solution and glucose solution. 10 µL sample solution (standard solution and calibration solution) was individually mixed with 1000 µL glucose solution, and then amalgamated under 37℃ for 10 min. Absorbance was observed under OD value 505 nm. For calculation of experimental results: Glucose content (mmol/L) = Absorbance of sample mixed solution (A)/Absorbance of calibration mixed solution (A) * Concentration of calibration solution (5.55 mmol/L). The sucrose content was determined using hydrolysate through a boiling water bath. The product has a maximum absorption peak at 290 nm, according to the OD value to calculate the content of sucrose. Measurement kit includes 10 µmol/mL sucrose standard solution and hydrolysate solution. 30 µL sample and sucrose calibration solution were mixed with 2 mL hydrolysate solution. Absorbance was observed under O.D. value 290 nm.

For calculation of experimental results: Sucrose content (µmol/mg) = (Absorbance of sample mixed solution—Absorbance of empty)/ (Absorbance of sample mixed solution—Absorbance of empty) * Concentration of standard solution (10 µmol/mg)*fold of dilution. The fructose content was measured through the product of the interaction between fructose and matrix fluid with a maximum absorption value at 285 nm. Measurement kit includes 1 mg/mL fructose standard solution and solution I. 50 µL samples and fructose standard solution were mixed with 3 mL solution I and boiled for 8 min. Absorbance was observed under O.D. value 285 nm.

For calculation of experimental results: Fructose content (mg/mL) = (Absorbance of sample mixed solution—Absorbance of empty)/ (Absorbance of sample mixed solution—Absorbance of empty) * Concentration of standard solution (mg/mL) *fold of dilution. The total soluble sugar content was defined using the colored reaction between sugar and enthrone. Measurement kit includes 100 µg/mL standard solution and substrate solution, and concentrated sulfuric acid didn’t supply. 200 µL sample and standard solution were mixed with 100 µL substrate solution and 1 mL concentrated sulfuric acid and boiled for 10 min. Absorbance was observed under O.D. value of 620 nm. For calculation of experimental results: The total soluble sugar content (µg/g) = (Absorbance of sample mixed solution—Absorbance of empty)/ (Absorbance of sample mixed solution—Absorbance of empty) * Concentration of standard solution (mg/mL)/fresh weight of samples (g)/volume of water * fold of dilution.

The root length of the plants was measured using a ruler, and then the average root length was calculated. Fresh weight was measured with a balance and the average weight was calculated.

### Yeast One-Hybrid

For one-Hybrid Library: The sugarcane cDNA yeast library materials were collected from the 60-day-old (seedling stage) *S. spontaneum* (SES 208), including mature leaf 1, roll leaf 1, stem 3, stem 6, stem 9, root and flower. The total RNA was extracted and the cDNA fragment was synthesized by reverse transcription after mRNA isolation. Using the Gateway method, cDNA was recombined into the pDONR222 vector by Gateway entry clone (BP reactions) to generate the primary cDNA library. The pGADT7 vector was then used for the expression clone (LR recombination reaction) to obtain the secondary library required for promoter binding examination. The yeast library was constructed by Ouyi Biomedical Technology Co., Ltd (Shanghai, China).

The procedures are followed by Matchmaker® Gold Yeast One-Hybrid Library Screening System User Manual (Clontech, Cat. Nos. 630491, 630,466, 630,499). Briefly, the 1999 bp *SsSWEET13c* upstream region (starting from ATG) was divided into five fragments (F0, F1, F2, F3 and F4). Primers used to amplify these fragments are listed (Additional file 3). The isolated shorter fragments of *SsSWEET13c* promoter were inserted into pAbAi vectors to construct the bait vector. After confirmation by sequencing and reconstructed vectors were linearized with restriction enzymes and create a bait/reporter strain by integrating the pBait-AbAi plasmid into the yeast competent cells Y1HGold yeast genome. Growth on the dropout medium (SD) lacking Uracil (-Ura) with AbA enables the determination of the correct concentrations to be used for screening the cDNA library. Construction and screening were conducted using a cDNA library by cotransformation and in vivo homologous recombination, grown on SD lacking leucine (-Leu) with AbA. The positive colonies were sequenced using the universal primer of pGADT7-F/R and then blasted against NCBI and PlantTFDB datasets.

### Analysis of the expression profiles of *SsSWEET13c* and potential TFs

The cDNA libraries for each sample were established according to the manufacturer’s protocol from Illumina TruSeq® RNA. Applied 100-bp paired-end sequencing via Illumina HiSeq2500 equipment at the Center for Genomics and Biotechnology (FAFU, Fuzhou, China). The raw data were aligned on the sorghum gene models using TRINITY (https://github.com/trinityrnaseq/trinityrnaseq/wiki) and the transcripts per million (TPM) value was calculated via the RSEM method.

## Supplementary Information

**Additional file 1. **Relative expression of overexpressing SsSWEET13c Arabidopsis thaliana lines by RT-qPCR. (A) Overexpression levels of 6 strains (B) Average overexpression levels of 6 strains.

**Additional file 2. **Root length and fresh weight of SsSWEET13c overexpression lines and WT.

**Additional file 3. **The fragment sequences used in yeast-one hybrid.

**Additional file 4.** PCR results of 1% agarose gel electrophoresis for *SsSWEET13c *promoter. (A) 1-2 indicated fragment (-1999 to -1721), 3-4 indicated fragment (-1580 to -1250). 5-10 were not used for this study. (B) 1-8 indicated fragment (-660 to -310). (C) 1-8 indicated fragment (-309 to -1). M, maker.

**Additional file 5.** The sequences of transcription factor from yeast one-hybrid.

**Additional file 6.** The sequences of transcription factor from yeast one-hybrid align to NCBI and PlantTFDB.

**Additional file 7.** Gene expression value based on transcripts per million (TPM).

**Additional file 8.** Pearson correlations between SsWEET13c and transcription factors expression pattern.

**Additional file 9.** RT-qPCR verification of nine TFs in partial segments of leaf gradients.

**Additional file 10.** Primers for amplifying the coding sequence of *SsSWEET13*.

**Additional file 11.** The primers for RT-qPCR of *SsSWEET13c* and nine TFs from yeast one-hybrid in *S. spontaneum*.

## Data Availability

All data generated during this study are included in this published article and its additional files. *S. spontaneum* gene sequence data are available in the accession numbers in Genbank: QVOL00000000, and the related RNA-seq data analyzed during the current study are also available in the Sugarcane database (SGD, http://sugarcane.zhangjisenlab.cn/sgd/html/index.html). The 20 TFs sequences were deposited into Genbank (accession numbers: ON929307-ON929326).

## Abbreviations

AbA: Aureobasidin
GAPDH: Glyceraldehyde-3-phosphate dehydrogenase
RT-qPCR: Quantitative reverse transcription PCR
TPM: Transcripts per million
SEs/CCs: Sieve element/companion cells
SWEETs: Sugars Will Eventually be Exported Transporters
TFs: Transcription Factors
WT: Wild-type
Y1H: Y1HGold: yeast one-hybrid yeast competent cells

## References

[1] Zhu XG, Long SP, Ort DR (2010). Improving Photosynthetic Efficiency for Greater Yield. Annu Rev Plant Biol.

[2] Stolz J, Stadler R, Opekarová M, Sauer N. Functional reconstitution of the solubilized Arabidopsis thaliana STP1 monosaccharide-H+ symporter in lipid vesicles and purification of the histidine tagged protein from transgenic Saccharomyces cerevisiae. Plant J. 2010;6(2):225–33. 10.1046/j.1365-313x.1994.6020225.x 7920712

[3] Lambers H, Chapin FS, Pons TL (2008). Photosynthesis. Plant Physiological Ecology.

[4] Ainsworth EA, Bush DR (2011). Carbohydrate Export from the Leaf: A Highly Regulated Process and Target to Enhance Photosynthesis and Productivity. Plant Physiol.

[5] Kühn C (2003). A Comparison of the Sucrose Transporter Systems of Different Plant Species. Plant Biol.

[6] Lalonde S, Wipf D, Frommer WB (2004). Transport mechanisms for organic forms of carbon and nitrogen between source and sink. Annu Rev Plant Biol.

[7] Sauer N (2007). Molecular physiology of higher plant sucrose transporters. FEBS Lett.

[8] Kühn C, Grof CPL (2010). Sucrose transporters of higher plants. Curr Opin Plant Biol.

[9] Chen LQ (2014). SWEET sugar transporters for phloem transport and pathogen nutrition. New Phytol.

[10] Wang L, Wang W, Wang YQ, Liu YY, Wang JX, Zhang XQ, Ye D, Chen LQ (2013). Arabidopsis galacturonosyltransferase (GAUT) 13 and GAUT14 have redundant functions in pollen tube growth. Mol Plant.

[11] Heikkinen S, Argmann CA, Champy MF, Auwerx J (2007). Evaluation of glucose homeostasis. Curr Protoc Mol Biol.

[12] Chen LQ, Hou BH, Lalonde S, Takanaga H, Hartung ML, Qu XQ, Guo WJ, Kim JG, Underwood W, Chaudhuri B (2010). Sugar transporters for intercellular exchange and nutrition of pathogens. Nature.

[13] Ge YX, Angenent GC, Wittich PE, Peters J, Franken J, Busscher M, Zhang LM, Dahlhaus E, Kater MM, Wullems GJ (2000). NEC1, a novel gene, highly expressed in nectary tissue of Petunia hybrida. Plant J.

[14] Lin IW, Sosso D, Chen LQ, Gase K, Kim SG, Kessler D, Klinkenberg PM, Gorder MK, Hou BH, Qu XQ (2014). Nectar secretion requires sucrose phosphate synthases and the sugar transporter SWEET9. Nature.

[15] Kanno Y, Oikawa T, Chiba Y, Ishimaru Y, Shimizu T, Sano N, Koshiba T, Kamiya Y, Ueda M, Seo M (2016). AtSWEET13 and AtSWEET14 regulate gibberellin-mediated physiological processes. Nat Commun.

[16] Chen LQ, Qu XQ, Hou BH, Sosso D, Osorio S, Fernie AR, Frommer WB (2012). Sucrose Efflux Mediated by SWEET Proteins as a Key Step for Phloem Transport. Science.

[17] Yuan M, Zhao J, Huang R, Li X, Xiao J, Wang S (2014). Rice MtN3/saliva/SWEET gene family: Evolution, expression profiling, and sugar transport. J Integr Plant Biol.

[18] Ma L, Zhang D, Miao Q, Yang J, Xuan Y, Hu Y (2017). Essential Role of Sugar Transporter OsSWEET11 During the Early Stage of Rice Grain Filling. Plant Cell Physiol.

[19] Manish K, Singh R, Singh O, Prakash S, Pandurang A, Madhu C, Debarchana J, Vineeta S, Diptibala R, Mukherjee A (2019). Genetic analysis for bacterial blight resistance in indica rice (Oryza sativa L.) cultivars. Oryza.

[20] Wu Y, Lee SK, Yoo Y, Wei J, Kwon SY, Lee SW, Jeon JS, An G (2018). Rice Transcription Factor OsDOF11 Modulates Sugar Transport by Promoting Expression of Sucrose Transporter and SWEET Genes. Mol Plant.

[21] Bezrutczyk M, Hartwig T, Horschman M, Char SN, Yang J, Yang B, Frommer WB, Sosso D (2018). Impaired phloem loading in zmsweet13a, b, c sucrose transporter triple knock-out mutants in Zea mays. New Phytol.

[22] Zhang J, Zhou M, Walsh J, Zhu L, Chen Y, Ming R (2013). Sugarcane Genetics and Genomics. Sugarcane: Physiology, Biochemistry, and Functional Biology.

[23] Hu W, Hua X, Zhang Q, Wang J, Shen Q, Zhang X, Wang K, Yu Q, Lin Y-R, Ming R (2018). New insights into the evolution and functional divergence of the SWEET family in Saccharum based on comparative genomics. BMC Plant Biol.

[24] Jin J, Zhang H, Kong L, Gao G, Luo J (2014). PlantTFDB 3.0: a portal for the functional and evolutionary study of plant transcription factors. Nucleic Acids Res.

[25] Li P, Ponnala L, Gandotra N, Wang L, Si Y, Tausta SL, Kebrom TH, Provart N, Patel R, Myers CR (2010). The developmental dynamics of the maize leaf transcriptome. Nat Genet.

[26] Emms DM, Covshoff S, Hibberd JM, Kelly S (2016). Independent and Parallel Evolution of New Genes by Gene Duplication in Two Origins of C4 Photosynthesis Provides New Insight into the Mechanism of Phloem Loading in C4 Species. Mol Biol Evol.

[27] Rolland F, Moore B, Sheen J (2002). Sugar sensing and signaling in plants. Plant Cell.

[28] Ni J, Li J, Zhu R, Zhang M, Qi K, Zhang S, Wu J (2020). Overexpression of sugar transporter gene PbSWEET4 of pear causes sugar reduce and early senescence in leaves. Gene.

[29] Klemens PA, Patzke K, Deitmer J, Spinner L, Le Hir R, Bellini C, Bedu M, Chardon F, Krapp A, Neuhaus HE (2013). Overexpression of the vacuolar sugar carrier AtSWEET16 modifies germination, growth, and stress tolerance in Arabidopsis. Plant Physiol.

[30] Meteier E, La Camera S, Goddard ML, Laloue H, Mestre P, Chong J (2019). Overexpression of the VvSWEET4 Transporter in Grapevine Hairy Roots Increases Sugar Transport and Contents and Enhances Resistance to Pythium irregulare, a Soilborne Pathogen. Front Plant Sci.

[31] Li P, Wang L, Liu H, Yuan M. Impaired SWEET-mediated sugar transportation impacts starch metabolism in developing rice seeds. The Crop J. 2021;10(1):98–108.

[32] Yang J, Luo D, Yang B, Frommer WB, Eom JS (2018). SWEET11 and 15 as key players in seed filling in rice. New Phytol.

[33] Sosso D, Luo D, Li QB, Sasse J, Yang J, Gendrot G, Suzuki M, Koch KE, McCarty DR, Chourey PS, et al. Seed filling in domesticated maize and rice depends on SWEET-mediated hexose transport. Nat Genet. 2015;47(12):1489–93. 10.1038/ng.3422 26523777

[34] Czechowski T, Bari RP, Stitt M, Scheible WR, Udvardi MK (2004). Real-time RT-PCR profiling of over 1400 Arabidopsis transcription factors: unprecedented sensitivity reveals novel root- and shoot-specific genes. Plant J.

[35] Jakoby M, Weisshaar B, Dröge-Laser W, Vicente-Carbajosa J, Tiedemann J, Kroj T, Parcy F (2002). bZIP transcription factors in Arabidopsis. Trends Plant Sci.

[36] Sakuma Y, Liu Q, Dubouzet JG, Abe H, Shinozaki K, Yamaguchi-Shinozaki K (2002). DNA-binding specificity of the ERF/AP2 domain of Arabidopsis DREBs, transcription factors involved in dehydration-and cold-inducible gene expression. Biochem Biophys Res Commun.

[37] Xu H, Wang X, Chen J (2010). Overexpression of the Rap2.4f transcriptional factor in Arabidopsis promotes leaf senescence. Sci China Life Sci.

[38] Wang Y, Henriksson E, Söderman E, Henriksson KN, Sundberg E, Engström P (2003). The arabidopsis homeobox gene, ATHB16, regulates leaf development and the sensitivity to photoperiod in Arabidopsis. Dev Biol.

[39] Irvine JE (1999). Saccharum species as horticultural classes. Theor Appl Genet.

[40] Zhang J, Arro J, Chen Y, Ming R (2013). Haplotype analysis of sucrose synthase gene family in three Saccharumspecies. BMC Genomics.

[41] Wang Y, Hua X, Xu J, Chen Z, Fan T, Zeng Z, Wang H, Hour A-L, Yu Q, Ming R (2019). Comparative genomics revealed the gene evolution and functional divergence of magnesium transporter families in Saccharum. BMC Genomics.

[42] Zhang Q, Hu W, Zhu F, Wang L, Yu Q, Ming R, Zhang J (2016). Structure, phylogeny, allelic haplotypes and expression of sucrose transporter gene families in Saccharum. BMC Genomics.

[43] Clough SJ, Bent AF (1998). Floral dip: a simplified method for Agrobacterium-mediated transformation of Arabidopsis thaliana. Plant J.

[44] Livak KJ, Schmittgen TD (2001). Analysis of relative gene expression data using real-time quantitative PCR and the 2(-Delta Delta C(T)) Method. Methods.

[45] Lescot M, Déhais P, Thijs G, Marchal K, Moreau Y, Van de Peer Y, Rouzé P, Rombauts S (2002). PlantCARE, a database of plant cis-acting regulatory elements and a portal to tools for in silico analysis of promoter sequences. Nucleic Acids Res.

[46] Shervedani RK, Mehrjardi AH, Zamiri N (2006). A novel method for glucose determination based on electrochemical impedance spectroscopy using glucose oxidase self-assembled biosensor. Bioelectrochemistry.

