# Supplementary material for: Functional characterization and analysis of transcriptional regulation of sugar transporter SWEET13c in sugarcane Saccharum spontaneum
Source: BMC Plant Biol. 2022 Jul 22;22:363. doi: 10.1186/s12870-022-03749-9 (PMC9308298; doi:10.1186/s12870-022-03749-9)
Supplement: Supplementary file 1 — Additional file 1. Relative expression of overexpressing SsSWEET13c Arabidopsis thaliana lines by RT-qPCR. (A) Overexpression levels of 6 strains (B) Average overexpression levels of 6 strains. [file 12870_2022_3749_MOESM1_ESM.pdf]

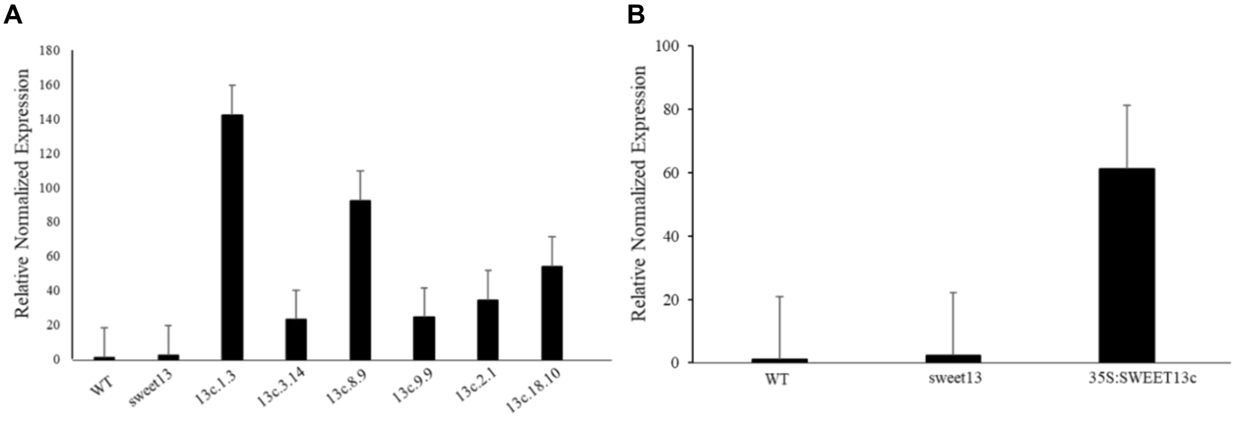


**Additional file 1: Relative expression of overexpressin**g ***SsSWEET13c Arabidopsis thaliana*** **lines by RT-qPCR**. (A) Overexpression levels of 6 strains (B) Average overexpression levels of 6 strains.
