# Supplementary material for: Functional characterization and analysis of transcriptional regulation of sugar transporter SWEET13c in sugarcane Saccharum spontaneum
Source: BMC Plant Biol. 2022 Jul 22;22:363. doi: 10.1186/s12870-022-03749-9 (PMC9308298; doi:10.1186/s12870-022-03749-9)
Supplement: Supplementary file 3 — Additional file 3. The fragment sequences used in yeast-one hybrid. [file 12870_2022_3749_MOESM3_ESM.pdf]

Additional file 3: The fragment sequences used in yeast-one hybrid.

***SsSWEET13c* promoter name (divided into 5 fragments)**	**Sequences**	**Primers**
SsSWEET13c fragment 0 (-1999 to -1721) F0	TTCAGTGGTCAAGAATCAAGATCGGCAATGAGCTT TTGAAAAGATAGCCATTTAACTGAAACCATTCTTTG CTACATCAATACCCATTTTAATCAACCACTTTGGTTT GCCTCCAATAATCGTGATTAGGATGGGGGGACGTA TGTGGCAAAAACGCATTCTCTCTCTTCCTTGTATCC ATCGGAAGCTACCGTCAAACTACATGGAAAAAACA TACTGTTTTCAGCAATTCTAGGACTTTAAACTACCA CTCTAGCCATATTATCACAATATGTGTCTA	F:TTCAGTGGTCAAGAA TCAA R:TAGACACATATTGTG ATAATATGG
SsSWEET13c fragment 1 (-1580 to -1250) F1	GACACATATTTTATGATCTAGATTAAGAGCACAGGA TGAAGGTGAAGATTTTTCACATGACAGCTTAAGTAA TTAATATCACATGTATGTTACTTAATTAGGAGCACG TGGAATCTTTAATATGTTTAATTATGCTTAGTGCTGT AATCATTGAATTGATTGGAAACCAAGCAATATAGAA AAAGAAAAGCTGGCGGAAAGCATTGTATGTCCACT AACAGCAGCGACGTGAGGAAAGTAGAATTAAAGAA GCAAAGGCATTCCAAAATGACCTATACATGCCGAC AAAGATCTATCCTGTTTTTGAGTCACTGTTACTGGT GCCTATATA	F :GACACATATTTTATG ATCTAGAT R :TATATAGGCACCAG TAACAGT
SsSWEET13c fragment 2 (-1218 to -707) F2	ATATATATATAGGGAGAGTATACTCTCTATAGCTAG CTACAAAATAAGTTATTTTGTAGCCACCTCTATTTAC GATAATTTTATATACTAATTTACGACAATGTTAGTAT ATATTTACGATAGTTGAATTACTACAACACATGGGG ATATTTACCATAATGTTATAGTAAATCACATAGTAAG GAGTTACTATAATCTCATAAATTAGTATACTAATTAT CGTAACTTAAAGTGGCTGCAGAATAAGTTATTTTGT AGCCAGCTACAGGGTAGTAGTTCTATATATATATAT ATATATATATATATATATTGCTATATTATTCAATTGAA GCAAAACAAGGATTTTTTAAAGCTGTAAAAAATATT TCATGGTGAAAACCTGAGTAATACACATAAAAAAAT GTTTAACCACCAGTGGTTAGAACAACTTAGTTTTAA TCTTTATGTCCAAATAAAAAGGATATTCTAACTTAAT TATTATTAGGTTCTACTCTGGAGCAAAATCACATA	F:ATATATAGGGAGAGTA TACTCTC R:TATGTGATTTTGCTC CAGAG
SsSWEET13c fragment 3 (-660 to -310) F3	ATGTGATTTAATTTGCTGTAGTGCGGCGGCTCTTAC CATTACCACTGAGTCGTCCAAAGTATGCTGGGGAG AGAACCCCAGGCGGTGCACAACAATTTGGAGTGTA CTACCCCTGGCACTCACCGTTGTATAATGGACTAT AAAGCATGCACGCAAAGCAGCTCCCGCTTTTTTGA ATGGAGAAGATTATCTCAATCTATTAATATATATAG GACAATTTATACACTCTAGTGATATATACATGGCCA GAAATTTGTCATCTCTAAAGCTATCCATATTGAAAA AGATCTTTGCTTTGCATATATAATTTCACAAAAAAAT ATTAATGACATATGTGAGATTGAAGAGCC	F:ATGTGATTTAATTTG CTGTAGT R:GGCTCTTCAATCTCA CAT
SsSWEET13c fragment 4 (-309 to -1) F4	CGCCTGCACATGTACACGATGAACCAAGGCCTGC GTGCCACCTCGTGCCTATATAAAGCCACCCACAGC CCTGCCATCATTGCAAGAGTTTCAGCCAACACAGA GCGAGGACTCCTTCTCACAACTCTCCCTTCCCTCC TGTAGGGCCAAAGGGTTAGAGAGGAAGAGAAGTT GTTCCCAAGCTAGCCTAACAAGAAAACAAGCTCAT CATTGTGATTCCAGCAACTGTTGGCTCAGTTGTATA GCTTGTGTTGGGAAATTCTTGTGACCCTATTATATT ATTTGTAGCTCCCCCTTTTCTTGTCGT	F:ATGTACACGATGAAC CAAGG R:ACGACAAGAAAAGG GGGAG
