# Supplementary material for: Functional characterization and analysis of transcriptional regulation of sugar transporter SWEET13c in sugarcane Saccharum spontaneum
Source: BMC Plant Biol. 2022 Jul 22;22:363. doi: 10.1186/s12870-022-03749-9 (PMC9308298; doi:10.1186/s12870-022-03749-9)
Supplement: Supplementary file 4 — Additional file 4. PCR results of 1% agarose gel electrophoresis for SsSWEET13c promoter. (A) 1-2 indicated fragment (-1999 to -1721), 3-4 indicated fragment (-1580 to -1250). 5-10 were not used for this study. (B) 1-8 indicated fragment (-660 to -310). (C) 1-8 indicated fragment (-309 to -1). M, maker. [file 12870_2022_3749_MOESM4_ESM.docx]

| **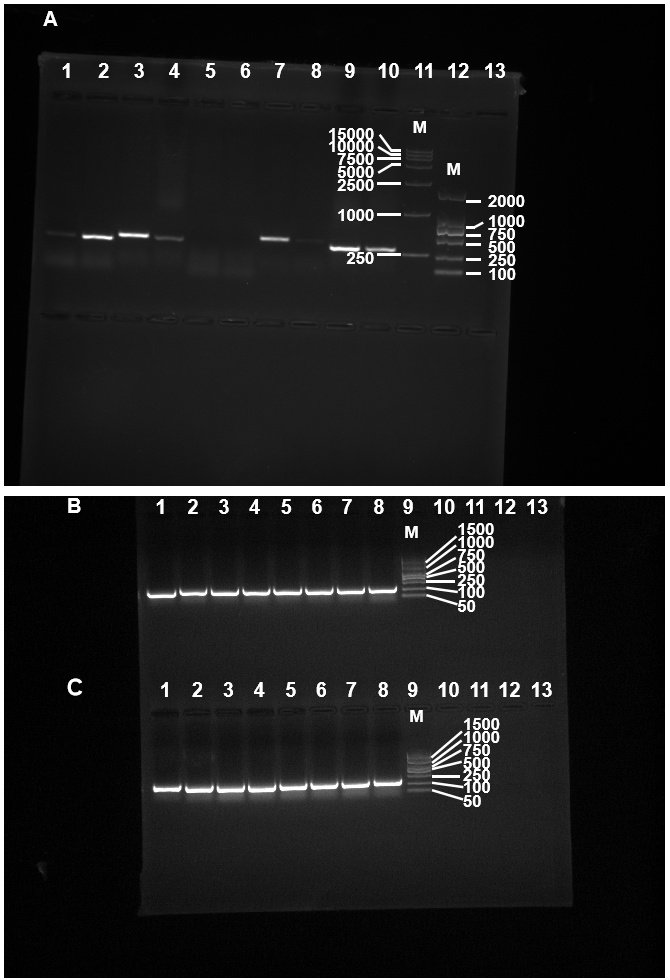** |
| --- |

**Additional file 4: PCR results of 1% agarose gel electrophoresis for *SsSWEET13c* promoter.** (A) 1-2 indicated fragment (-1999 to -1721), 3-4 indicated fragment (-1580 to -1250). 5-10 were not used for this study. (B) 1-8 indicated fragment (-660 to -310). (C) 1-8 indicated fragment (-309 to -1). M, maker.
