# Supplementary material for: Functional characterization and analysis of transcriptional regulation of sugar transporter SWEET13c in sugarcane Saccharum spontaneum
Source: BMC Plant Biol. 2022 Jul 22;22:363. doi: 10.1186/s12870-022-03749-9 (PMC9308298; doi:10.1186/s12870-022-03749-9)
Supplement: Supplementary file 5 — Additional file 5. The sequences of transcription factor from yeast one-hybrid. [file 12870_2022_3749_MOESM5_ESM.pdf]

Additional file 5: The sequences of transcription factor from yeast one-hybrid.

>13c-0-1

GGCCGGGGGGGATCCCATACGACGGTACCAGGATTACGCTCATATGACAAGTTTGTACAA
AAAAGTTGGCTCTCCTGGGAAGTCGGCAGCCCTGGGCGGAGTCGCTGGAAGGCGTGC
GGCGTGGCCTCTCCGGCCGCATCAGCCGGCCGACGGGCACCGCCCGCGTGGCCTTTC
CCTGCTTCTCTTCTTCTGATCTGATTTCATATATATACGTGTACCCCGTAGATGTAGATGTTG
GCACACGAAGTGGTAGAACTGTATAGATTAGCCTGCATTTCTCTGATTTGAAGAACGTATA
CGAATACAACTTGCTATTTTCAATTTGACATGGAAGTCCTCGGTAGGTGTCACCGGATCCA
ATTTGAGAGCCCATTATAAAGCACAGGTACAGAAGAGTAGTAGACGATGAAATTTAAGATTT
ATCATGACATTTCCCTCATTTGAAACTGAACATTCTTGCTCAACAATATTGACATATGCATTG
CTTGATTGCTTCTTCTATGCAGTAATTACAAAGTTTTCAATCCAGCCAGCTACAGCTTCCTG
TAATGCATAGGCGATAATGACATTGAGCACACCAAAATAGAAGCCTGCCTGACACTGAAAC
TGCCCATGGATGATGAGTCCAAGGAGCGAGAAGCCAGGAGGTGTTGTTGTGGCAGATGC
TGGAGTCAAGAGCCAGCGTTGTGCGGACAAGCATGCAGGAACTGAGAGTCATCGTCCTC
CTGTGGCGGACGAGCTAGAAGGTGCCATCATGCGTGGAATAGAAGTAGGAGGACCAGGT
TGAAGGCCTTGGGGAGATTTCCTGGAATGACTTTGTAGTTGCCATCATGGACCGGAAATA
ACATGTACGTTTATGTGCTATCCAAGTCACTGTTAACAACTTCAGTGACTTAGTGTAGTGAA
TGTTTGTACATATGACACTATCCTTTTAATGTAACAGTTGGGAATTACTTATGGATGTGTTGG
AATGAGATTTGGCTTTAAAAAAAAAAAAAAAAAAAAACCCCCTTTTCTTTGAAAAAGGGGGG
CTCGAGCTGCAGATAAAATCGAAAAATCTGAAAACCCCCCCCAAGGTTACACAA

>13c-0-2

GTTGGCCGGGGATCCCATACGACGTACCAGATTACGCTCATATGACAAGTTTGTACAAAAA
AGTTGGAAACCACGCCAACTCAAACCCTGCGAATTCCTCGCGGGCATCGGCAACGGGCA
ACGGCGATGGCGGCGTCCCCCTCTTCCGTCGCCGAAGTAGTAGTGGCCGCTGCTGCTG
CGGCAGCGATGGCGTCAGCTTTGCCGCAGGAGCAGCAGCAGCATCAGCAGCAGGCGC
GACCGCGGCCGCGGCCGATGCACGCGGGGGGCGGTGGCGCCAATGGCCGCCACCAC
GCGTACAGCCGCAAGGACAAGTCGCTCGGCCTCCTCTGCTCCAACTTCGTGGTTCTCTA
CAACCGCGACGACGTGGAGTCCATCGGGCTGGACGAAGCGGCCAAATGCCTCGGCGTG
GAGAGGCGCCGGATCTACGACATTGTCAACGTGCTCGAGAGCGTCGGGATTCTTGTGAG
GAAAGCCAAGAATCGCTACACCTGGATAGGTTTCGGCGGAGTCCCAATGGCCTTGCGAG
AACTCAAGGAGAGGGCGTTAAGGGAGAAGTCTGGCTTGGCTCCTCTGCAAACCGAGCA
GCAATCTGCTGCTACTATTTCTGATGATGAAGATGATGATACATTGGGCAATCCGGGTGCT
GATATCGAGAACGAGAAACTGAGTCAGACTGTGGACAATCTTTCTGACAAACCTGGTGCA
CCTCGTTGCCGCCTTAGATCTGATCATAGGAAGGAAAAGTCACTTGGGTTGCTCACACAG
AATTTCGTGAAGCTCTTCCTCACCATGGAGGTTGACACGATCTCACTTGATGAAGCTGCA
AAGCTGCTCCTTGGGAGAAGGTCACGAAGAGACCAATATGAGAACTAAAGTTCGGAGGTT
ATATGACATTTGCCAATTGTCCTGTCTTTCCCTGACTCTAATGTGAAGATACTAGCAAGTTG
ACTTCAGGGAGGCTGCTTTTTCGTGTGGTTCGGTAGGCCAAAATGCCGAATACAAGAAAA
TTGTGGTAACAGCTGCTTCTCCTCCCACCCACTGAAGT

>13c-0-5

AGCGCGCGCTGGGCCCCCCTCCGACGTACCAGATTACGCTCATATGACAAGTTTGTACAA
AAAAGTTGGACTCCTCTCTTCTCCTCACATATGCTGGTAGGCGACGGCGGCGACGGACT
CACGCCGGTGGTTGAGGCCGAGGGCGCACTGCAGTGGGTAGGTCCACGCGACGACGG
CGCGGGGCTGAAGGCTCCTCCCGCGGCGCGACGCCTCAGCGCGCAGAGCTGGGGAG

CAGCGGTGCTGCAGCTCCGTGCGCTCGAGAAGGACAGCGACCATCGCTGGATCCATCG
AGGAGGGTGCGGGATCCGGCGCAAGGAGGCAGCGGCAAATGAGATCCACGTGTGTGG
CGTCTCGTGGTGCTCCCCCAGCAGCGGCACGGGCGCTCCTCTCCTGGGAAGTCGGCA
GCCCTGGGCGGAGACGCTGGAAGTAATTACAAAGTTTTCAATCCAGCCAGCTACAGCTTC
CTGTAATGCATAGGCGATAATGACATTGAGCACACCAAAATAGAAGCCTGCCTGACACTGA
AACTGCCCATGGATGATGAGTCCAAGGAGCGAGAAGCCAGGAGGTGTTGTTGTGGCAGA
TGCTGGAGTCAAGAGCCAGCGTTGTGCGGACAAGCATGCAGGAACTGAGAGTCATCGTC
CTCCTGTGGCGGACGAGCTAGAAGGTGCCATCATGCGTGGAATAGAAGTAGGAGGACCA
GGTTGAAGGCCTTGGGGAGATTTCCTGGAATGACTTTGTAGTTGCCATCATGAACCGGAA
ATAACATGTAGGTTTATGTGCTATCCAAGTCACTGTTAACAACTTCAGTGACTTAGTGTAGG
TGAATGTTTGTACATATGACACTATCCTTTTAATGTAACAGTTGGGAATTACTTATGGATGTG
TTGGAATGAGATTTGGGAAAAAAAAAAAAAAAAAAAACCCCCTTTTTCTTTAACAAAAGGGG
GTGCCGCGCGCAGAAAAAAGCAAAAAAGCGAAAACCCGCGCGGAATGCACAAAAG
>13c-0-6
GAGTGAACCATACGACGTACCAGATTACGCTCATATGACAAGTTTGTACAAAAAAGTTGGA
CCAAAAGGTCTCTTCGGCGGCGGCGGCGGCGATGTCTCTTCCCCCGGGCCCGGCGGA
CGGCGGCACCGGCGATGACTGGTTCCTCGACTGCGGCATCCTCGACGACCTCCCGGCC
GCGGCCTGCGGGGCCTTCCCGTGGAACGCGTCCCCGTCTTCTTCCAACCCCAGTGTGG
AAGTGGGCAGCTATGTGAACACCACTGATGTCTTCAAGGAGCCCAATGATGTCTTCAAGG
AGCCTGGCAGCAATAAACGTTTAAGGTCAGGATCCAATGATGTGCCAACATCTAAAGCTTC
TAGGGAAAAAATGAGGAGGAACAAACTGAATGACAGGTTTCTTGAATTGGGGTCTACATT
AGAACCTGGGAAGCCAGTAAAAGCTGACAAAGCTGCTATCCTAAGTGATGCTACTCGCAT
GGTTATTCAGCTTCGTTCAGAAGCACAGCAGCTGAAGGAAACTAATGGTAGTCTTGAAGA
AAAGATTAAAGAACTAAAGGCCGAGAAGGATGATCTTCGTGTTGAGAAGTGGAAGTGGTG
TTCTCACATAAAAGTTTATTCCCCTTTATTCATTCTTATGATGTGTTATCTGGTCAAAATGCAT
CTTCCTTCCCAGAGTTCCCCTGATTACCTTACGCGTCCCAGCGCACGCCGCCATGACTGT
GGACAGCGCTTTGGCCCAGACGCCGTAGCGTTATTTCTGTTTTTGCTCTGTTTGACGTAG
AGCATAGATATGAAAGTGTCCAAGTATGATTTCAGAG
>13c-0-7
GGGCGGTGTACCATACGACGTACCAGATTACGCTCATATGACAAGTTTGTACAAAAAAGTT
GGAGTGGATGGTTGTGATAGGAGGTTCAGTATGAAGGCAAATATGCAGCGGCATGTTAAG
GAAATTCATGAGGATGAAAATGCTAGTAAGAGCAACCAGCAGTTTATTTGTAAGGAGGAG
GGCTGCAACAAGGTGTTGAAGTATTCATCAAAGCTGAAGAAACATGAGGAATCCCATGTT
AAATTGGACTATGTGGAGGTATTGTGCGGTGAACCTGGCTGCATGAAGATGTTTACAAAC
GTTGAATATCTGAGGGTTCATAACCAATCTTGCCATCAGTATATTCAGTGTGAGATATGTGG
AGTAAAGCACCTGCTCTGGTTCGTCAAGTGCTCTTTGTTGTCCCTTGACTATGACTCCATT
GGCATACTCTTGGTTTTCTTTATTAACAGAAGCGTTTGCTCTATCTTTTTCTTTTGCTAAATG
ATCACGCCTAATAGTTGGGGTTTTTC
>13c-0-8
GGGCCTTCCGACGTACCACATTACGCTCATATGACAAGTTTGTACAAAAAAAATGGAGCAA
GATGCCACAAGTGCTTGAATGGACTTCCATCAGGCGCTGGAGACCCTCCTGAGAAGCTC
CTTCAGGAGTGCCTTGTTCACGCGCTTCTTCCGAGGCCATTTTCCTGCACTGTTGATGGT
TGCCCCTTGAGCTATAGAAGGAAGGATCATTTGAACAGACATTTACTTACTCGTGAAGGGA
AACTATTTGTGTGCCCTATTGAAGGATGTGGCCGTGAGTTCAATATCAAGGGTAATATGCA

GAGACATGTTCAGGAAATCCACAAAGATGGCTCTCCTTGTGAAAGGAACAAAGAATTCAT
CTGTCCAGAGGTTAACTGTGGGAAGACTTTCAAATATGCTTCCAAGTTAAAGAAGCACGA
AGAATCACATGTTGAGCTGGAATACACAGAAGTTATCTGCTGTGAACCAGGTTGCATGAAA
TTCTTTTCAAACACAGAATGCCTCAAGGCGCATAACCAATCCTGTCATCAGCATGTTCCGT
GTGATATCTGTGGCACGAAACAGCTAAAGAAGAATTTCCAGCGCCATCGTCTGATGCATG
AAGGTTGCTGCCTCACTGATACGGTTAAATGCCACTTCGAGGACTGCAAATGTTCATTTTC
AAAGAAATCCACTTTGGACAAGCATGTTAAGGGGTCCATGAGCAGCGTAGGCCTTTTGTA
TGCCAATTCTCTGGGTGTGGAAGAGATTTTTCTTACAAGCATGTAAGGGACAATCATGAGA
AGTCAAGCGCTCATGTGCCACTGAGGGTGATTTTGTCGAGGCTGATGAGCAGCGACCAC
GCTCAGTAGGGTGGACGAAGAGGAAATCTGTATCTGTTGAGAGTCTGATGCGGAAGAGG
GTAGCTGCTCCTGATGATGGGCCTGCTCATGCTGATGGAACTGAGTATTTGAGGTGGCTT
CTATCAGGTTGATCCTCCTGGACCCGAAAGGAATAGATGTAGATACGCTCAAACCACCTTC
TTGAAGTTGGAACCTC
>13c-0-9
TGGGGCCGGGGGGGGCCACCCCCACGACCCCCATAACGCTCATATTACAAGTTTGTACA
AAAAAGTTGGAATGTACCGCGGCGTGCGCCAGCGCCACTGGGGCAAGTGGGTGGCGGA
GATCCGCCTCCCCAAGAACCGCACCCGGCTGTGGCTCGGCACCTTCGACACCGCCGAG
GACGCGGCGCTCGCCTACGACAAGGCCGCCTTCCGCCTCCGCGGCGACATGGCGCGC
CTCAACTTCCCGGCCCTCCGCCGCGACGGCGCGCACCTGGCCGGCCCGCTCCACGCC
TCCGTGGACGCCAAGCTCACTGCCATCTGCCAGTCCCTGGCGGGGTCCAAGAACGGCT
CCTCCGGCGACGAGTCGGCCGCGTCCCCGCCGGACTCCCCCAAGTGCTCGGCGTCAA
CGGAGGGTGAGGGGGAGGAGGAGTCGGGCTCCGCCGGCTCCCTTCCTTCCCCGACGC
TGACGCCGCCCGTGCCGGAGATGGCGAAGCTGGACTTCACCGAGGCGCCGTGGGACG
AGACGGAGACCTTCCACCTGCGCAAGTACCCGTCCTGGGAGATCGACTGGGATTCCATC
CTCTCATGAACGATCAACTACAACTGCTATAGTAGTAGCAGCAGCAAGATTCAGTCAGTGT
CAGCTCAATGATAGCTCTGTGTAATTTTCGATTTGGGGTTTGCAGCTGCGGTGGCTCGAT
GGCATTTTAGACATCGGCCATGGCGGCTGCGAGTAGCAATGAGTAACTAGCTAGTACATC
GTCGTCCAGTGTTGTGATGCAGCAGTAAGTACGTGCTAATCTCCTGGTTGAGCTGCCGGT
TGTTTTTTCTCACGGCACGGCCAGTCGAGAAAGTCAGTGTAATCCCGTGTTATTTAGTGCT
ATGATCTATCTGTTGCAGCTTAATTAAGTCCTCGAGCTTATGTAAAAAAAAAAAAAGACCCC
CTTTTCTTTTGCAAAAATGTGGCTGGACTCGCAATAGATCTAAAATACTGAAAAACCCGCC
GCCAGATGTCAACAAATGGTGTCTTGATTGACGAGTATCAAATCCTCTTAGTTGAGAGACA
CGTAGTC
>13c-0-11
AGGTAGTACCATACGACGTACCAGATTACGCTCATATGACAAGTTTGTACAAAAAAGTTGG
ACCGAGCTCCACGGCAAACAAGCGGAAGAGAAGGCAACAAGCCTACGAGCGGCGACGC
CAGTTGGATCGTTGTTCCATCCATGGCGCCCAAGAGATCGACGTCGCCGGCCGGAAGCA
GCAGCAGCGGTGGCAGTGGAAGCGGCGTGTCCGTAGCGGCGGCTGCGGCGGAGCAG
CCGAGGCTGCGCGGCGTGCGGAAGCGGCCGTGGGGCCGGTACGCGGCGGAGATCCG
GGACCCGGTGCGGAAGGCGCGCGTGTGGCTGGGCACCTTCGACACGCCCGAGCAGGC
GGCGCGGGCGTACGACGCCGCCGCGCGCAGGCTCCGCGGGACCGGTGCCATCACCAA
CTACCCCGCCGCCCCGGAGCCGTTGGCGGAGCCGGCTCCAGCGCCGAGCGGCAGCG
GGAGCGCCGTGCTGTACGAGTCGGCGTCGTCGGCGTCCTCGTGCTCCCTTCTGCCGGA
GTCGGTGACGGTGGCGGTCGCGGCGCCGCCTCCGTCGCTGGACCTCAGCCTGGCGCT

GCCGACTGCGGCGGCCGCGCAGACGTACCAGCTGTTCATGGACCCGACGGCGGCCGT
GACGCCGGCGCTGCTGCAGTTCCTGCCGCCGAAGAGCGAGGAGGAGCAGAGCTGCTC
TGGGTCGTCGCCATCGTCGTCTGTGGTGTTCGACGCGGCGGCGCCGCCCGTGGGCCT
GGGGCTGGACCTCAACCTGGCGCTGCTGCCGGCCGAGATGGTCATGTGAATTGTGATTG
CCGCTGTCCAGGAGACTCTAATCGATTGCTATATTAATTAGCCATTGTTAGTAGTAGTAGCT
AATAGGCACTCGTTCAGGTATAATAGGATGCAGATTTGTGCATGTCACTTGTGCATGCGCG
TAGCCCTGTTTTCTTTGTACTGACAGATTTGCATCTTTGCTGCAAGTCACGCAACAATGAA
AAAAAAAGGAGCATTATTATGCCCCCCAAGAAAAAAAAAAAAATAAAAGATAAAGAAAGAA
AAACCCCCCTCTTTTTTTTTATAAGAGGTGTGGCCGCCGCCGAGTATAGATCTCTCTCATG
AACAACACCGCCACAGACGACACGGAAGAAGGGTGGC
>13c-0-12
GGGCCTTGCATACCATACGACGTACCAGATTACGCTCATATGACAAGTTTGTACAAAAAAG
TTGGAGCAACCCGCACGAACCCTAGCTGGCGGCGGGAGGCGGAGGTGGCAGCGGCGG
CGGGATGACGCGGCGGTGCTCGCACTGCAGCCACAACGGGCACAACTCGCGGACGTG
CCCCAACCGCGGGGTCAAGATCTTCGGGGTGCGCCTCACCGATGGCTCGGCCATCCGC
AAGAGCGCCAGCATGGGGAACCTCTCCCTCCTCTCCGCGGGATCCACCAGCGGCGGCG
CGTCCCCCGCGGACGGCCCCGACCTCGCCGACGGCGGCGCCGGGGGCTACGCCTCC
GACGACTTTGTCCAGGGCTCCTCCTCCGCCAGCCGCGAGCGCAAGAAAGGTGTTCCTT
GGACTGAAGAAGAACACCGGAGGTTTTTGCTGGGATTACAAAAGCTTGGGAAAGGTGAT
TGGCGAGGAATTTCTCGGAATTTCGTGGTCTCAAGAACACCTACTCAAGTAGCAAGTCAT
GCTCAAAAATATTTTATACGTCAATCAAATATGAGCAGAAGGAAGAGAAGGTCTAGCCTTTT
TGACATGGTGCCTGATGAGTCCATGGACCTTCCACCCCTTCCTGGAAGTCAAGAACCAG
AGACCTCAGTGTTAAATCAACCACCACTGCCGCCTCCTGTGGAGGAGGAGGTGGAATCG
ATGGAGTCAGATACTTCTGCTGTTGCAGAGAGTTCTGCAGCTTCTGCTGTCATGCCCCGA
GAGTTTACAGCCTACCTATCCGATGATTGTTCCAGCTTATTTCTCACCGTTTTTGCAATTCT
CAGTTCCTTTCTGGCCAAATCAGGAAGATGGAGGCGATATGGCCCAAGAAACACACGAG
ATTGTCAAGCCTGTCTCAGTTCATTCCAAGAATCCAATTTATGTTGATGAACTTGTGGACAT
GTCAAAGCTAAGCATAAAGGAGCCGTGTCAGGAGACAGCGTTACTTCTCTGTCGATAAAT
CTACTAGGGGGTCAAAAATAGGCAGTCGGCTATCATGCAATCCTCAAACGAGAAAGCTCA
AGCCTGATTCTCAATCACCACTGAAACTGCATTTAGAATGGAAATCCTGGGGTGAAAATAG
CTTGCCTCTTTTTTTTTCATTATTTAAGATATACCGTGTTCTGAACTGTACCCATGGAGCGT
TGAAGTAAG
>13c-0-15
GGGGCGGGGGGCCCATACGACGTACCAGATTACGCTCATATGACAAGTTTGTACAAAAAA
GTTGGAGCTCGGGGCTCTTTCTCCCTTGTGCTAGCGGTTGCAGCGGTGGCTTCTAAATC
CATCCATCCTTGTGGCTTTGTGACTTGTGAGAGCTCGAGAGAAAGGATCGGAGATCACC
GCCTGCTACGGGGGCGCCACGGGGGGGGATGGACAGCCTGATCGAGGTGCACCTGGG
CGCGACGAAGCTCCAGACCATGCTGGAGGAGTCACCCACGCCTTCGATCGCGGCCGCA
GCGGGGACCACCAGTAAGGTCAGACTGACGCTTGACGGGATGATGAGCCGCCTGTCGA
GCGCCATGTCGGCTCTGGATACCACCGGAGCCGGCGCCGCCGCCAGCCATCAGCCACC
GGGCCGGAGGAGGAGGAGAGGCGGCGCGGCCGGCGCAGTACGTCGCCTTTGCGGAC
TTGGACTCCACGCTTCCCACATCCCCCTGACTGAATCCAGCCCCACGAAAAGCAGCTAAT
CCGCCTCAACTGCGCCTGCTTTATCTTAAATGTAAGCTTATAGGTTTGTTCAGTTGCTGATT
ATTAAGAAAATGTTTGACTCTTCCTCCGATCAATATACATAATTTAGCAAATGATCAAAACTC

CTGATAAAGTGCACCGATAACAACAAAACTAATAAGACAAGAGTATGTACTATCGCTCGGC
CTCCTCTGTCTGGAATAAAACGTAACTCACCAACGACAAGAGTTTCATACCGCTGATAACC
CGTATGATAAACACTTTGTTTGATCCACTTTATGTAGCCACCA
>13c-0-16
GGGGCGCGTGTATTCCATACGACGTACCAGATTACGCTCATATGACAAGTTTGTACAAAAA
AGTTGGACTGCTGCTGCCTCGAGTGAGTCACTGAGTGAGCTGTGGACCTGTGGTGGTC
GTGATCTTCCATTCTTCCCTCTTCCCTCTTCCCTCGCCACTTGCTGGGTTGTTGTGTGGC
CATGGCGGCAGCGGCGGCGCCTTTCGCGCAGGTGATGGAGGACATGGCGAAGGGCCA
GGAGTACGCCACGCAGCTGCAGGCGCTGCTCCGGGACTCGCCCGAGGCCGGCCGCCT
CGTCGACCGGATCCTCCACGCCATGTCCCGCACCATCGACACCGCCAAGGCGGCCGCC
GCCGAGGAGGAGGCGTCCGAGGTGCAGAGCGACGTCACCTGCGCCGGCACTGCCGCC
GGCGGCAAGCGGAAGGCCGCTGCTGGTGGAGGGGACAAGAGGGCCGCCTGTCGGAA
GAGAGGCCAGCAATCGTCGGTTGTGACGAAGAACACCAAGGATTTGGACGACGGACAC
GCATGGCGCAAGTACGGACAGAAGGAGATACAGAACTCCAAGCACCCAAAGGCCTACTT
CCGGTGCACGCACAAGCACGACCAGCAGTGCTTGGCGCAGCGGCAGGTGCAGCGCCG
CGACGACGACCCGGACGTCTACACTGTCACCTACATCGGCGTGCACACCTGCCGGGAC
CCGGCCACCGCCGTCGGGCCGCTCGTTGTGCACGCGGCCGCCCAGGAGCTCCACCAC
GCGGGGTCCCGCCTCATCAGCTTCGCGGCCAACGCCAGCGTCGCCACGACCAGCACCA
CCACCACCGGGAACACCACCGGCCAGCAGGCCGGGCACAAGGACGCCGCCGCGCTGC
TGCTGGCGAGGCCCCTGAAGCTGGAGGGCGGCGGCGAGCAGGAGGAGGTGCTGAGCA
GCCTCACCCCGGCGCCCGGCCCGGACCAGACGGACGTGACGTCCGGCCTGCAGCTGC
AGCAGCACCACTGCTACTGGCGGTGACCTCGCGGACATGGACGGCCGCACTTCGGG
>13c-0-17
GGCACAGCATACCATACGACGTACCAGATTACGCTCATATGACAAGTTTGTACAAAAAAGT
TGGATCCGCATCTTGTTCTGCTGCTACTCCTGTCCGCCCCGGTCAGGCTCGACTACTCG
CCGATCTCAGGAGACCCGCCCACAACATGGGCTGCCCAAGCCGACGACGACCTGAAGG
GCAGGAGGCTCCGCCTCCCGCTGCCAGGTCCCCGCCCGCGGGACCACCAAGTCGGGG
CTGACAACAGTACCACCCTGGACCTGCTCCCGGTAAAGGGCCCCAACGGCGGGTTTTCT
GAAGATCCGCCCCCACCATAACACGCTGCCGCTGCTGATCCGGAAGCTGCCCTTCCGGA
CCTGGTCCGCGAGATCGCCCAGGACTTCAAGACGGACCTCCCGGCTGCAAGCCTCCCG
TGCTGGGATGTCCAGGAGGCCGCCGAGGCCTACCTAACGGGACTCTTCCAGGACACCT
GCCTGTGCGCCATACAGATGCTGAGCGTCACCATCATGCCCAAGGACATCCAGCTCGCG
CGCCGCATGCTGGGCCAGCGCACGTAAGCGTGACTGAACTCCCTGCGTCCCGGCGGAG
GTGGGCACTGAGGGATGTCAATTTGGAACCTGTTCGCTTGTTGATCTCCTGCCGCGGATA
GTGTATCTGATGAATCTGATGATGTTAAATGTGTAAAGACTGATGTAGCCTCGGTGGGTGA
GTTGATTATTGTCGTAAAACAGCTTTACAAGATGTATTATGTGCTCGTTGCTACTTTGTTTG
GAGTCATCATCGTGTCAAATGCAATCCTGGATATAGATACTATGACATAACAATATTACCAGT
ATGCGCTACTAAAAACGGATTGCTACACACAGTCCCTAGCTTTCATGGGAGATCCGGTGA
CTGCACGGCATAATTTATGAAACAAAAAAATTCTAACAACCCTCACAGAAGAATACGCCCT
GATGGTCGATTAACATGGATTGCTTTACGCTAAGCAAATGACTAACC
>13c-0-18
TGGCGGGTACCATACGACGTACCAGATTACGCTCATATGACAAGTTTGTACAAAAAAGTTG
GGTCGAGCTAATTGAGTGGGAGCTAGAAAAACCCAGAGGACGAGCAGCGAGCGACTCG
GCAGCGAGCGCCGTCCTGTGTGCGGCCGGTGTGTCTGCGCTAGAGGGTGAACGGGAT

GGATCACCTTCGTCAATAATTGCGGTACGAACACGCAAATCCCATAGCTTATTAAGAACTC
GCTGCCGTTACTGCTGGTAGCGAGAGAAATAAGGCAGCAGAAGAATTGCGGAGGGCCG
GGATCGTCGATTTGGAGAGGGAAGCCTGCGTGGGCGGTGACTGATCGAGAGATCAGCT
GACCACCATGGGGCGCGGGAAGGTGGTGCTGCAGCGGATCGAGAACAAGATCAGCCG
GCAGGTGACCTTCGCCAAGCGCCGGAACGGCCTGCTCAAGAAGGCCTACGAGCTCTCC
ATCCTCTGCGACGCCGAGGTCGCGCTCGTCCTCTTCTCCCACGCCGGCCGCCTCTACCA
GTTCTCATCCTCCTCCAACCTGCTTAAGACTCTAGAGCGGTACCAGAGGTACATCTATGCT
TCAGCTGATGCTGCAGTGCCATCTAGCGATGAGATGCAGAATAACTATCAAGAATATGTGA
AGCTGAAGGCACGAGTTGAGGTTTTACAACACTCACAAAGGAATCTTCTGGGTGAAGATC
TGGCTCCACTTGGCCCTAGTGAACTTGATCAGCTTGAGAGTCAAGTAGACAAGACCTTGA
AGCAGATAAGATCAAGAAAGACTCAAGTGTTACTTGATGAACTTTGCGACTTAAAGAGAAA
GGAACAAATGCTGCAAGATGCAAACAGGGTTCTGAAAAGGAAGCTGGACGAATTCAAGG
CAGAGGCTGCTTCTCCCCCGCAGCTAGCGTGGCAAGATGGAGGCGGCATGTTGTCCCAT
GACCCTCCACAGCCAGAACACTTCTTCCAGGCTCTGGAGAGCAACCCATCTCTGCAACC
TACATACCATACGATGGACATGAACCAGGAGCCAGT
>13c-0-20
ACCCATACGGACGGTACCAGGATTACGCTCATATGACAAGTTTGTACAAAAAAGTTGGCC
GACGCGAAGAACGGAAGAGCGGCGGCGGCGGCGTTGACGCGGGGAAGAGTTGCTCTA
AGGCTGAGCAGGAGCGGCGCAAGGGCGTCCCGTGGACGGAAGAGGAGCACAGGCTGT
TCTTGCTGGGTCTGGACAAGTTCGGCAAGGGCGACTGGCGGAGCATCTCGCGCAACTT
CGTCATCTCACGGACGCCGACGCAGGTGGCGAGCCACGCACAGAAGTACTTCATCCGC
CTCAACTCCATGAACCGCGACCGGCGCCGCTCCAGCATCCACGACATCACCAGCGTGAC
CGCCGGCGATGTCGCCGCGGCCGGCGCACCCATCACGGGCGGGCCAGCCGCCGCGG
GGGCGATGCCGATGGGGCCTGCGGGCATGAAGCACCACCACCCGGCTCCGCCGATGG
GCATGTACGGGCACGCGCCCATGGGCCACCCAGTCGCGGGGCACATGGTGGCGCCGG
CGGCCGTGGGCACGCCCGTCATGTTCCCGCCCGGTCATCACTCGCCCTATGTCGTGCC
AGTGGGATACCCGGCGCCACCGGCCAAGATGCACCAATGATCGATCGTCTACGGGCGG
CATTTGCACATCTGCAGAGCTGCATTTCGTTGCTTGTTCTTTCTTGATTTCGTTCATCTTTG
TGTAGCCAAAGCCATCTATATATCCATCCTTTCCTGATCTTCCGGCCCTTTGATTTAACATA
GATGATTTGAGTGAGTGAGTGCAGGGCAGGCAGGCGATGAGGTGAATAATGGGTATCTG
CTGACTGTTGCAATAGCGCTACTTATTGTAATCACACTTAGGGAGGCTCAAGGAATGTGTA
ATTAGTAGCAGCCTTCCAGGGTGCAAATGTTGCAATTGCAACTGGGGACTGATCCGATTC
CATTTTCCGGATGTAAATTGATTGTCTATAAAATCTTATCTGTGCTGTATTGCAAGAAATATA
AGACCCCCTATTTTCTTTAAAGGGGTGGCCCCGCTGCGAAAAAAAACACGAAGAACTCAG
AACACCCGCGAGGTGGTGAAAAAGAAA
>13c-0-22
TCGTACAAAAAAGTTGGGCCGCCGGCGGTAGGAGTGGGTGGTGTCAGGGGAGGAGGA
GACGGCGGGGCCGGCTATGTCTCCGATGATCCCGGGCACGCAACCTGCTCGACGAATG
GCCGCGTCGAGCGCAAGAAAGGTACACCTTGGACTGAAGAAGAGCATATAATGTTTCTGA
TGGGTCTTCAGAAGCTTGGTAAGGGAGATTGGCGCGGAATATCTCGAAACTTTGTTGTTT
CCAGGACCCCGACTCAAGTGGCAAGCCATGCTCAAAAGTACTTTATTAGACAGACAAACT
CATCAAGACGGAAGAGGCGGTCAAGCTTGTTTGACATGGTTGCAGAAATGCCAATGGAC
GAGTCTCTAGCTGCTGCAGAACAAATTACTGTCCAAAATACTCAAGATGAAGCTACAATTT
CAATTCAACTGCCAACCTTACATCTTGAGCAACAGAAGGAAGCAGAGTTTGCTAAGCAAC

TGCCAACTTTTCAGCTAAGGCAGCATGAGGAATCTGAATATGCAGAACCTTCATTGACACT
ACCAGTTTTGGAGATGAACTCCAGCGTACCATTCAAGACCATAGCTGTTCCGCCGGTACC
AGCATTCTACCCTGCATTGGTCCCTGTTCCATTAACTCTTTGGCCTCCAAGTGTTGCCCAT
GTGGAGGAAGCAGGCACAACCCATGAAATCCTAAAACCAACTCCTCTGAATGGTAAGGA
GGTGATTAAGGCAGATGACGTTGTTGGTATGTCTAAGCTCAGCATTGATGAGGCCAGCTC
TGGCTCCATGGAACCCACTGCTCTTTCCCTTCAGCTTATTGGATCGACAGATACAAGGCA
GTCAGCTTTTCATGTGAGTCCACCAATGAATAGACCTGAACTAAGCAAGAGAAACCGCAG
TCCAATTCATGCCGTTTGAGGAAGCATGGAACAGGACCACTGGGTCAAGTCTTGCAGATT
ATACTATTGGTCGCACTATCCCAGAATGTGAGCATGTACTCTG
>13c-0-24
TGGGGGGTCCCCTTCCCACGTACCAGATTACGCTCATATGACAAGTTTGTACAAAAAAGT
TGGACTTCCGGCACACAGGATACCACCGTAGACAGATTCGTCGCGACCATGGGGCTCGA
CGTCGTGGAGATCGGGATGGGCGCCGATTTGAGCCTGGATCTGAGGCACTTCGCCTCC
AAGGCCGTGAGGCAGAGCAAGGACGACGCGCCGGCGCCGGACATGGACGCCTGCATC
CGCCGCCTCGAGGAGGAGCGGGGCAAGATCGAGATGTTCAAGCGGGAGCTCCCGCTCT
GCGCGCGGCTCCTCGCCGACGTGATTGATGTCATGAAGGAGGAGGCGGGGAAGAAGAC
GACGCGGAGGAGTGATCGCAGCCTGGCGGCTGCGGCGGCGGTGGCGGAGGACGAGG
AGGACGGCGCCGCCGGGGACAAGAGCAAGTGGATGAGCACGGCGCAGCTCTGGACGG
GCGATTCGGGCCGGCAGGACGCGGAATCAGAGAAGCAAGACAAGGGGAGGAGCTCGC
CGTCCCGCGGCGGCGCCGGCGGCGGTATCTTGCCGTTCAAGGCTGCTGTGGGCTCCG
GCGCGCCGGCGTTCGCGCCGGTCTGCTTGAGAATGGACGACAAGGCTGCGCGCGTCG
GGATGCCGGATCTGTCCTTGATGTCGCCGCCGGCGACCAAGAGCGCCGGCGAGGATAG
CCGGCGCCAGGTGGTGGGGTTTGCGCAGGCAGCGGCGAGGGCGGCCGCCATGCCGA
CCGCTGCCCCTGCGCAGTCCCAATCGCAGCAGCAGCAGTTCAAGGAAGGTTCGGCGCT
GCTGGGCGACGGATCTGCCTTCGCCAGTTCGTCGCCGCCTTGAATCAACTCGGATGGAC
CCCGAGGTTTGCCACCCCAACGCATATCTGGGAGCTTATGAAGGGGGAAGGGCGGAGA
AAAGGAGCAGGAAAAGCTCATCGCCGAACACCCGGCTGCACAACTGATTGCCCCGGGAT
TCCGAACGAGATCAGACAC
>13c-0-26
TGGAACGGTTACCAGGATTTACGCTCATATGACAAGTTTGTACAAAAAAGTTGGTCCGAG
CCTCGGCCTCGGCCTCGGCCTCCGACGGCTTCCATTGCACGCTGGCGAATGGCGACCT
TCAAATCAAACTGACAAATTGATTGGAGCAAAACCGAAGTGGAAGTATAGATAGATTCTCT
AATTCGAGAAAATTGCATATTTGGGACTCTAATTCCCCTCCCGCTTCTCAGAAATCGCTGA
GTTTTTTTCTTTTACCTTTGGGAGGGAAGAGGCGGCGGCGTTTGTTTAGCAGGGGAAGAT
CCCCTTCCGCAATCATCCAACGGGCGCCACCATCCAGATCAGTTTGGAGCATGGGGAGT
AAATCTGTTCCATCCCGCTTCGTGGCCACATGAAACCACTATGTATAATCCCAAAAGGAAC
ACGAAGCAGGTGGTTTCTCCATTGCCATGGAACAGCCAACCACTGAAGAGCAGAAAGGA
CATTCAGTATTAAGCTTAATTGGTGGCTGATAACTGCTAGGTTTAGCATCAGTCGAAAGCA
CAAGCCCTGGCTCTGCTGGAATGCTCCCTCCTTGCAGAAGCATGTGCCTTTCTGGAGGG
AATGTCTGCCTAGAAGACAGGAAATGGCTGTGGCTCTGAAGCTGTTGTTGATGGTGATAC
ATCTGGCATGAAGACATTGCAGGCTTAAAGTTTATCGGCCCTTTGTTTTTTGGTGAACTAA
GGGTTCTTTGTCACACAATGGCGTTATATTCCTTGGGGAATGCTCTAAGAAACTATGACTA
CTCATGCCAAGATGTTATGCAAAGTCTGGTTTTATAAGGAAGGCAAGTAAGGAAGGCAATC
AACGTGCCTGAACCGTGACCTCTTTTTGTCTTGTGCTCTTTTTGTCCCTGTATGTGTCTCC

CTGTTTTCTCGTCTCTTTTGGGTTTCATCTCTAGCCTACCCCAACTTGCTTGGGACAAAAG
GCTATGTTGTTGTTGTTGTTGTTGTTTGCAAGTGAAGGAAAGAATTTCGAATCGTTAGAAA
CAAAACACTCAAGCCTGGTCCAATTCTTGGCGGCAACTGAAAAGAA

>13c-1-2

GGCACAGCATACCATACGACGTACCAGATTACGCTCATATGACAAGTTTGTACAAAAAAGT
TGGATCCGCATCTTGTTCTGCTGCTACTCCTGTCCGCCCCGGTCAGGCTCGACTACTCG
CCGATCTCAGGAGACCCGCCCACAACATGGGCTGCCCAAGCCGACGACGACCTGAAGG
GCAGGAGGCTCCGCCTCCCGCTGCCAGGTCCCCGCCCGCGGGACCACCAAGTCGGGG
CTGACAACAGTACCACCCTGGACCTGCTCCCGGTAAAGGGCCCCAACGGCGGGTTTTCT
GAAGATCCGCCCCCACCATAACACGCTGCCGCTGCTGATCCGGAAGCTGCCCTTCCGGA
CCTGGTCCGCGAGATCGCCCAGGACTTCAAGACGGACCTCCCGGCTGCAAGCCTCCCG
TGCTGGGATGTCCAGGAGGCCGCCGAGGCCTACCTAACGGGACTCTTCCAGGACACCT
GCCTGTGCGCCATACAGATGCTGAGCGTCACCATCATGCCCAAGGACATCCAGCTCGCG
CGCCGCATGCTGGGCCAGCGCACGTAAGCGTGACTGAACTCCCTGCGTCCCGGCGGAG
GTGGGCACTGAGGGATGTCAATTTGGAACCTGTTCGCTTGTTGATCTCCTGCCGCGGATA
GTGTATCTGATGAATCTGATGATGTTAAATGTGTAAAGACTGATGTAGCCTCGGTGGGTGA
GTTGATTATTGTCGTAAAACAGCTTTACAAGATGTATTATGTGCTCGTTGCTACTTTGTTTG
GAGTCATCATCGTGTCAAATGCAATCCTGGATATAGATACTATGACATAACAATATTACCAGT
ATGCGCTACTAAAAACGGATTGCTACACACAGTCCCTAGCTTTCATGGGAGATCCGGTGA
CTGCACGGCATAATTTATGAAACAAAAAAATTCTAACAACCCTCACAGAAGAATACGCCCT
GATGGTCGATTAACATGGATTGCTTTACGCTAAGCAAATGACTAACC

>13c-1-3

GGGGCATGCGTACCATACGACGTACCAGATTACGCTCATATGACAAGTTTGTACAAAAAAG
TTGGGTTACGGCGTGGTCGGGATGGAGGCGGACGCGGACGAGGAGATGATGGCGTGC
GGCGGCGGCGGCGGAGGTGGGGAGAAGAAGCGGCGCCTGAGCGCGGAGCAGGTGCG
CGCGCTGGAGCGAAGCTTCGAGGTGGAGAACAAGCTGGAGCCGGAGCGCAAGGCGCG
GCTGGCGCGCGACCTGGGCCTGCAGCCGCGCCAGGTCGCCGTCTGGTTCCAGAACCG
CCGCGCCCGCTGGAAGACCAAGCAGCTGGAGCGCGACTACGCCGCGCTCCGCCACTC
CTACGACGCGCTACGCCACGACCACGACGCGCTCCGCCGCGACAAGGACGCCCTCCTC
GCCGAGGTACTGTTGTCGATGTTGTGCCGTTGTATAAGCTGCTGGCCGGACGAGCACGA
TTAAGGTGTTGTTTGTTTCTTCATTTACGTGCGCAACGCAATGACTGATCCTAACTCGGTT
GGTTGTCTCAATTGTCGTAGATCAAGGAGCTCAAGGCGAAGCTAGCGGACGAGGAGGCA
GCGGCGAGCTTCACGTCGGTCAAGGCGGAGCCGGCTGCCTCGGACGGGCCTCCCCCC
GTCGGGGTGGGGTCGTCCGAGAGTGACTCCAGCGCGGTGCTGAACGACGCGGACCCG
CCCGTGCCGGAGGCGCCGGTGCCGGAGGTACAGGGTGGTACTCTTCTTGACGCGCCCT
GCCCCGTGGCCGGCCCAGCGGTGGCGGTGAACCATGGCGGGGTATTCTTCCACGGGA
GCTTCCTGAAGGTGGAGGAGGACGAGACGGGGCTCCTGGACGACGACGAGCCCTGCG
GCGGGTTCTTCGCCGTGGAGCAGCCGCCGCCGATGGCGTGGTGGACCGAGCCCACGG
AACACTGGAACTGAACCGAGGCAGGCAGGATGATGCAGTGCAGCGTAGACTCGGGATCT
GGGCCGAGCGGTGGAGGAATAAAGAATTCGGAGCGAAACACGGA

>13c-1-5

TGGAACGGTTACCAGGATTTACGCTCATATGACAAGTTTGTACAAAAAAGTTGGTCCGAG
CCTCGGCCTCGGCCTCGGCCTCCGACGGCTTCCATTGCACGCTGGCGAATGGCGACCT
TCAAATCAAACTGACAAATTGATTGGAGCAAAACCGAAGTGGAAGTATAGATAGATTCTCT

AATTCGAGAAAATTGCATATTTGGGACTCTAATTCCCCTCCCGCTTCTCAGAAATCGCTGA
GTTTTTTTCTTTTACCTTTGGGAGGGAAGAGGCGGCGGCGTTTGTTTAGCAGGGGAAGAT
CCCCTTCCGCAATCATCCAACGGGCGCCACCATCCAGATCAGTTTGGAGCATGGGGAGT
AAATCTGTTCCATCCCGCTTCGTGGCCACATGAAACCACTATGTATAATCCCAAAAGGAAC
ACGAAGCAGGTGGTTTCTCCATTGCCATGGAACAGCCAACCACTGAAGAGCAGAAAGGA
CATTCAGTATTAAGCTTAATTGGTGGCTGATAACTGCTAGGTTTAGCATCAGTCGAAAGCA
CAAGCCCTGGCTCTGCTGGAATGCTCCCTCCTTGCAGAAGCATGTGCCTTTCTGGAGGG
AATGTCTGCCTAGAAGACAGGAAATGGCTGTGGCTCTGAAGCTGTTGTTGATGGTGATAC
ATCTGGCATGAAGACATTGCAGGCTTAAAGTTTATCGGCCCTTTGTTTTTTGGTGAACTAA
GGGTTCTTTGTCACACAATGGCGTTATATTCCTTGGGGAATGCTCTAAGAAACTATGACTA
CTCATGCCAAGATGTTATGCAAAGTCTGGTTTTATAAGGAAGGCAAGTAAGGAAGGCAATC
AACGTGCCTGAACCGTGACCTCTTTTTGTCTTGTGCTCTTTTTGTCCCTGTATGTGTCTCC
CTGTTTTCTCGTCTCTTTTGGGTTTCATCTCTAGCCTACCCCAACTTGCTTGGGACAAAAG
GCTATGTTGTTGTTGTTGTTGTTGTTTGCAAGTGAAGGAAAGAATTTCGAATCGTTAGAAA
CAAAACACTCAAGCCTGGTCCAATTCTTGGCGGCAACTGAAAAGAA
>13c-1-7
GGGCGGTGTACCATACGACGTACCAGATTACGCTCATATGACAAGTTTGTACAAAAAAGTT
GGAGTGGATGGTTGTGATAGGAGGTTCAGTATGAAGGCAAATATGCAGCGGCATGTTAAG
GAAATTCATGAGGATGAAAATGCTAGTAAGAGCAACCAGCAGTTTATTTGTAAGGAGGAG
GGCTGCAACAAGGTGTTGAAGTATTCATCAAAGCTGAAGAAACATGAGGAATCCCATGTT
AAATTGGACTATGTGGAGGTATTGTGCGGTGAACCTGGCTGCATGAAGATGTTTACAAAC
GTTGAATATCTGAGGGTTCATAACCAATCTTGCCATCAGTATATTCAGTGTGAGATATGTGG
AGTAAAGCACCTGCTCTGGTTCGTCAAGTGCTCTTTGTTGTCCCTTGACTATGACTCCATT
GGCATACTCTTGGTTTTCTTTATTAACAGAAGCGTTTGCTCTATCTTTTTCTTTTGCTAAATG
ATCACGCCTAATAGTTGGGGTTTTTC
>13c-1-11
GGGGGGGGTCCCATACGACGTACCAGATTACGCTCATATGACAAGTTTGTACAAAAAAGT
TGGCAAGGACTTCGGGTCCATGAACATGGACGAGCTCCTCCGCAGCATCTGGTCCGCG
GAGGAGATACACAACGTCGCGGCCGCCAACGCGTCGACGGCGGCGGACCACGCCGCA
TGGGGGTCGTCCATCCAGCGCCAGGGCTCGCTCACCCTCCCCCGCACGCTCAGCCAGA
AGACCGTCGACGAGGTCTGGCGCGGCCTCGTGTGTGTCGGCGGCGGACCCTCCGCCG
AGGCTGCGGCGCCGCCCCCACCGGCCCAGCGGCAGCCCACGCTCGGGGAGATCACGC
TGGAGGAGTTCCTCGTCCGTGCCGGCGTGGTGAGGGAGGACATGACGGCGCCGCCGC
CCGTACCGCCGGCGCCGGTGTGCCCGCCGCCTCCTCCGAGGCCGCAAATGCTGTTTCC
CCATGGCAATGTGTTTGCTCCCTTGGTGCCTCCGCTGCAATTCGGGAATGGGTTGGTGT
CGGGTGCTGTCGGTCAGCAGCAGGGAGGTGGTCCTGCGGCCCCGCCGCGGCCGGTGA
CGGCCAGCGGGTTCGGGAAGATGGAAGGAGACGAGTTGTCGTCTCTGTCGCCATCACC
GGTGCCGTACGTTTTCGGTGGTGGTTTGAGGGGAAGGAAGCCACCAGCTATGGAGAAG
GCGGTTGAGAGGAGGCAGCGCCGGATGATCAAGAACCGGGAGTCCGCCGCGAGGTCG
CGTCAGAGGAAACAGACATATATGATGGAGCTGGAAGCTGAGGTGGCAAAACTTAAAGAG
CTGAACGATGAATTGCACAAGAAGCAGGTCGAAATGTTGGATAAGCAAAATAATGAGGTC
CTGGAGAGAATGAGACGGCCAGTTGGACCCACAGCAAAGATAATTTGTCTGCGGAGGAC
ATTGACAGGTCCGTGGTATGCTGTACACCTGTGGGGCTGTACCTAGCAGTATCACTCGTG
AAGGGAGTGTATATATAGTAGGTGAACTCGACCCCTATGGCCATAGTTGTCCTGGTTGACC

CAGCTTATTAATCCAGAGCTTCATCGTAAGGTGATAGATGGCCTGTGGATTTCCCCCAGT
>13c-1-13
GGGGGGGTACCCCTACGACGTACCAGATTACGCTCATATGACAAGTTTGTACAAAAAAGT
TGGCCGAGCTCCCTTGCTAGGTTACCTTCCTGAACCACATTCTGCCCTTCAGGCCCTCAT
CCCTCTCACTCTCTCCTAGTCTCTTCAGTAACAGCACAATTTTGTTATTGACACACCTTCAA
TCTGTTGACCTGCTACTAGCAATAACTGGAGCTCCAACCTTTCTGTCTCTGAAACTTGGA
GTTCCCAAGGGGAGGCTCTGCCAAATTTCAGCTTCTGAACATCCATTTTAAGGGGGAGGT
TCTGCCAAAATTTCGGCTTCAGAACATCCATTTTCAGGGGGAGGTTCTGCCAAAATTTGC
TCCTGTGAACATAAGTTTCCCAAGGCGAGGTTCTGCCAAAATTTCCACCTGTGACCATCA
GTTTTCCAGGGGGAGATTCTGCCAAAATTTCCACCTGTGACACATCATTGATACATACAAT
AAAATCTAATTTGCAAGGTAAATCAGCTGAAGGACCAGAACAAGCAGCTGAGCATGGCAT
TGAGCATAACCAGCCAGAACCTTGTGGCAGTGCAAGCACAAAACTCTGTCCTGCAGACC
CAGAAGATGGAGCTGGACAGCAGGCTGGGTGCCCTGACAGATATCCTCTGGTACATGAA
CTCAAGCACCAGCACCAGCACTGCTCCTACAAATCCAGCCATGGTGAATGGCTTCACAAC
ATGGAGCAGTGCCTCTGATATTCTTGGCGCCAGCGCATGGAACCAGCAGCAGCCCATAG
ATCTGTACCAATGCTTCTAGCTCTAGCTCTAGAAGAGAGAAGGTGGTTGTGGCATATGACA
TAGTGGTAGGGCCAGGAGGAAAGGAGGCAGGCAGGCATCAAGTTTCTGTTCAATCATGT
AGGGCCTTTGTGTCACTATTGATGTGTGCTTTGATGCTCCTCCTTCATTCATTGTTATTTTG
GGAGGCGACGGTGATGTTTTGTTTTCTTGCTGTTGTGTAAAGCCTAAAGATGACAAATGG
TAAAGTTGTCTTGTTGTGTGTGACTGAAGCGTTGTCTCTGGTTGTGAGAATATATGT
>13c-1-14
GGCCGGGGGGGATCCCATACGACGGTACCAGGATTACGCTCATATGACAAGTTTGTACAA
AAAAGTTGGCTCTCCTGGGAAGTCGGCAGCCCTGGGCGGAGTCGCTGGAAGGCGTGC
GGCGTGGCCTCTCCGGCCGCATCAGCCGGCCGACGGGCACCGCCCGCGTGGCCTTTC
CCTGCTTCTCTTCTTCTGATCTGATTTCATATATATACGTGTACCCCGTAGATGTAGATGTTG
GCACACGAAGTGGTAGAACTGTATAGATTAGCCTGCATTTCTCTGATTTGAAGAACGTATA
CGAATACAACTTGCTATTTTCAATTTGACATGGAAGTCCTCGGTAGGTGTCACCGGATCCA
ATTTGAGAGCCCATTATAAAGCACAGGTACAGAAGAGTAGTAGACGATGAAATTTAAGATTT
ATCATGACATTTCCCTCATTTGAAACTGAACATTCTTGCTCAACAATATTGACATATGCATTG
CTTGATTGCTTCTTCTATGCAGTAATTACAAAGTTTTCAATCCAGCCAGCTACAGCTTCCTG
TAATGCATAGGCGATAATGACATTGAGCACACCAAAATAGAAGCCTGCCTGACACTGAAAC
TGCCCATGGATGATGAGTCCAAGGAGCGAGAAGCCAGGAGGTGTTGTTGTGGCAGATGC
TGGAGTCAAGAGCCAGCGTTGTGCGGACAAGCATGCAGGAACTGAGAGTCATCGTCCTC
CTGTGGCGGACGAGCTAGAAGGTGCCATCATGCGTGGAATAGAAGTAGGAGGACCAGGT
TGAAGGCCTTGGGGAGATTTCCTGGAATGACTTTGTAGTTGCCATCATGGACCGGAAATA
ACATGTACGTTTATGTGCTATCCAAGTCACTGTTAACAACTTCAGTGACTTAGTGTAGTGAA
TGTTTGTACATATGACACTATCCTTTTAATGTAACAGTTGGGAATTACTTATGGATGTGTTGG
AATGAGATTTGGCTTTAAAAAAAAAAAAAAAAAAAAACCCCCTTTTCTTTGAAAAAGGGGGG
CTCGAGCTGCAGATAAAATCGAAAAATCTGAAAACCCCCCCCAAGGTTACACAA
>13c-1-15
GGGGGCGGGCATACATACGACGTACCAGATTACGCTCATATGACAAGTTTGTACAAAAAA
GTTGGACTAGCCTCGCCTCGCCCCGACCGCGGAATCCCCCCCCGGCCGCCGCGACCG
CGACCGCGACGATGGACTGCGCCGTAGGCGGAGACCCCGTGGAGGACTTCCTCCTCGG
CGGCGCCGGCGACGACGGGGATCTCGCCATCTTCTGCGACGGAGGACTCGGGATTGAG

GGTGTCAATGGAGATGCTTGTGGATTTGACCAATCTAATTTAGGGAAAAGGGGTAGAGAT
GAACCGTCTTCATCTGGTCCAAAATCCAAAGCTTGTCGTGAAAAAATGAGGAGGGACAAG
CTGAATGACAGGTTCCTGGAATTAAGTTCAGTTATGAATCCTGGTAAACAAGCAAAGTTGG
ATAAAGCAAACATCTTGAGTGATGCAGCCCGTATGGTGGCGCAACTGAGAGGTGAGGCA
GAAAAGCTTAAGGAATCAAATGACTAGCTGCCGGAGACTGGTTGTACATGGATCAAAAAA
GTCCGATCACTCCAATGATCTCTTT
>13c-1-16
TGGGGCCGGGGGGGGCCACCCCCACGACCCCCATAACGCTCATATTACAAGTTTGTACA
AAAAAGTTGGAATGTACCGCGGCGTGCGCCAGCGCCACTGGGGCAAGTGGGTGGCGGA
GATCCGCCTCCCCAAGAACCGCACCCGGCTGTGGCTCGGCACCTTCGACACCGCCGAG
GACGCGGCGCTCGCCTACGACAAGGCCGCCTTCCGCCTCCGCGGCGACATGGCGCGC
CTCAACTTCCCGGCCCTCCGCCGCGACGGCGCGCACCTGGCCGGCCCGCTCCACGCC
TCCGTGGACGCCAAGCTCACTGCCATCTGCCAGTCCCTGGCGGGGTCCAAGAACGGCT
CCTCCGGCGACGAGTCGGCCGCGTCCCCGCCGGACTCCCCCAAGTGCTCGGCGTCAA
CGGAGGGTGAGGGGGAGGAGGAGTCGGGCTCCGCCGGCTCCCTTCCTTCCCCGACGC
TGACGCCGCCCGTGCCGGAGATGGCGAAGCTGGACTTCACCGAGGCGCCGTGGGACG
AGACGGAGACCTTCCACCTGCGCAAGTACCCGTCCTGGGAGATCGACTGGGATTCCATC
CTCTCATGAACGATCAACTACAACTGCTATAGTAGTAGCAGCAGCAAGATTCAGTCAGTGT
CAGCTCAATGATAGCTCTGTGTAATTTTCGATTTGGGGTTTGCAGCTGCGGTGGCTCGAT
GGCATTTTAGACATCGGCCATGGCGGCTGCGAGTAGCAATGAGTAACTAGCTAGTACATC
GTCGTCCAGTGTTGTGATGCAGCAGTAAGTACGTGCTAATCTCCTGGTTGAGCTGCCGGT
TGTTTTTTCTCACGGCACGGCCAGTCGAGAAAGTCAGTGTAATCCCGTGTTATTTAGTGCT
ATGATCTATCTGTTGCAGCTTAATTAAGTCCTCGAGCTTATGTAAAAAAAAAAAAAGACCCC
CTTTTCTTTTGCAAAAATGTGGCTGGACTCGCAATAGATCTAAAATACTGAAAAACCCGCC
GCCAGATGTCAACAAATGGTGTCTTGATTGACGAGTATCAAATCCTCTTAGTTGAGAGACA
CGTAGTC
>13c-1-18
AGGTAGTACCATACGACGTACCAGATTACGCTCATATGACAAGTTTGTACAAAAAAGTTGG
ACCGAGCTCCACGGCAAACAAGCGGAAGAGAAGGCAACAAGCCTACGAGCGGCGACGC
CAGTTGGATCGTTGTTCCATCCATGGCGCCCAAGAGATCGACGTCGCCGGCCGGAAGCA
GCAGCAGCGGTGGCAGTGGAAGCGGCGTGTCCGTAGCGGCGGCTGCGGCGGAGCAG
CCGAGGCTGCGCGGCGTGCGGAAGCGGCCGTGGGGCCGGTACGCGGCGGAGATCCG
GGACCCGGTGCGGAAGGCGCGCGTGTGGCTGGGCACCTTCGACACGCCCGAGCAGGC
GGCGCGGGCGTACGACGCCGCCGCGCGCAGGCTCCGCGGGACCGGTGCCATCACCAA
CTACCCCGCCGCCCCGGAGCCGTTGGCGGAGCCGGCTCCAGCGCCGAGCGGCAGCG
GGAGCGCCGTGCTGTACGAGTCGGCGTCGTCGGCGTCCTCGTGCTCCCTTCTGCCGGA
GTCGGTGACGGTGGCGGTCGCGGCGCCGCCTCCGTCGCTGGACCTCAGCCTGGCGCT
GCCGACTGCGGCGGCCGCGCAGACGTACCAGCTGTTCATGGACCCGACGGCGGCCGT
GACGCCGGCGCTGCTGCAGTTCCTGCCGCCGAAGAGCGAGGAGGAGCAGAGCTGCTC
TGGGTCGTCGCCATCGTCGTCTGTGGTGTTCGACGCGGCGGCGCCGCCCGTGGGCCT
GGGGCTGGACCTCAACCTGGCGCTGCTGCCGGCCGAGATGGTCATGTGAATTGTGATTG
CCGCTGTCCAGGAGACTCTAATCGATTGCTATATTAATTAGCCATTGTTAGTAGTAGTAGCT
AATAGGCACTCGTTCAGGTATAATAGGATGCAGATTTGTGCATGTCACTTGTGCATGCGCG
TAGCCCTGTTTTCTTTGTACTGACAGATTTGCATCTTTGCTGCAAGTCACGCAACAATGAA

AAAAAAAGGAGCATTATTATGCCCCCCAAGAAAAAAAAAAAAAATAAAAGATAAAGAAAGAA
AAACCCCCCTCTTTTTTTTTATAAGAGGTGTGGCCGCCGCCGAGTATAGATCTCTCTCATG
AACAACACCGCCACAGACGACACGGAAGAAGGGTGGC
>13c-1-19
TGGGGGGTCCCCTTCCCACGTACCAGATTACGCTCATATGACAAGTTTGTACAAAAAAGT
TGGACTTCCGGCACACAGGATACCACCGTAGACAGATTCGTCGCGACCATGGGGCTCGA
CGTCGTGGAGATCGGGATGGGCGCCGATTTGAGCCTGGATCTGAGGCACTTCGCCTCC
AAGGCCGTGAGGCAGAGCAAGGACGACGCGCCGGCGCCGGACATGGACGCCTGCATC
CGCCGCCTCGAGGAGGAGCGGGGCAAGATCGAGATGTTCAAGCGGGAGCTCCCGCTCT
GCGCGCGGCTCCTCGCCGACGTGATTGATGTCATGAAGGAGGAGGCGGGGAAGAAGAC
GACGCGGAGGAGTGATCGCAGCCTGGCGGCTGCGGCGGCGGTGGCGGAGGACGAGG
AGGACGGCGCCGCCGGGGACAAGAGCAAGTGGATGAGCACGGCGCAGCTCTGGACGG
GCGATTCGGGCCGGCAGGACGCGGAATCAGAGAAGCAAGACAAGGGGAGGAGCTCGC
CGTCCCGCGGCGGCGCCGGCGGCGGTATCTTGCCGTTCAAGGCTGCTGTGGGCTCCG
GCGCGCCGGCGTTCGCGCCGGTCTGCTTGAGAATGGACGACAAGGCTGCGCGCGTCG
GGATGCCGGATCTGTCCTTGATGTCGCCGCCGGCGACCAAGAGCGCCGGCGAGGATAG
CCGGCGCCAGGTGGTGGGGTTTGCGCAGGCAGCGGCGAGGGCGGCCGCCATGCCGA
CCGCTGCCCCTGCGCAGTCCCAATCGCAGCAGCAGCAGTTCAAGGAAGGTTCGGCGCT
GCTGGGCGACGGATCTGCCTTCGCCAGTTCGTCGCCGCCTTGAATCAACTCGGATGGAC
CCCGAGGTTTGCCACCCCAACGCATATCTGGGAGCTTATGAAGGGGGAAGGGCGGAGA
AAAGGAGCAGGAAAAGCTCATCGCCGAACACCCGGCTGCACAACTGATTGCCCCGGGAT
TCCGAACGAGATCAGACAC
>13c-1-22
GGGCCTTCCGACGTACCACATTACGCTCATATGACAAGTTTGTACAAAAAAAATGGAGCAA
GATGCCACAAGTGCTTGAATGGACTTCCATCAGGCGCTGGAGACCCTCCTGAGAAGCTC
CTTCAGGAGTGCCTTGTTCACGCGCTTCTTCCGAGGCCATTTTCCTGCACTGTTGATGGT
TGCCCCTTGAGCTATAGAAGGAAGGATCATTTGAACAGACATTTACTTACTCGTGAAGGGA
AACTATTTGTGTGCCCTATTGAAGGATGTGGCCGTGAGTTCAATATCAAGGGTAATATGCA
GAGACATGTTCAGGAAATCCACAAAGATGGCTCTCCTTGTGAAAGGAACAAAGAATTCAT
CTGTCCAGAGGTTAACTGTGGGAAGACTTTCAAATATGCTTCCAAGTTAAAGAAGCACGA
AGAATCACATGTTGAGCTGGAATACACAGAAGTTATCTGCTGTGAACCAGGTTGCATGAAA
TTCTTTTCAAACACAGAATGCCTCAAGGCGCATAACCAATCCTGTCATCAGCATGTTCCGT
GTGATATCTGTGGCACGAAACAGCTAAAGAAGAATTTCCAGCGCCATCGTCTGATGCATG
AAGGTTGCTGCCTCACTGATACGGTTAAATGCCACTTCGAGGACTGCAAATGTTCATTTTC
AAAGAAATCCACTTTGGACAAGCATGTTAAGGGGTCCATGAGCAGCGTAGGCCTTTTGTA
TGCCAATTCTCTGGGTGTGGAAGAGATTTTTCTTACAAGCATGTAAGGGACAATCATGAGA
AGTCAAGCGCTCATGTGCCACTGAGGGTGATTTTGTCGAGGCTGATGAGCAGCGACCAC
GCTCAGTAGGGTGGACGAAGAGGAAATCTGTATCTGTTGAGAGTCTGATGCGGAAGAGG
GTAGCTGCTCCTGATGATGGGCCTGCTCATGCTGATGGAACTGAGTATTTGAGGTGGCTT
CTATCAGGTTGATCCTCCTGGACCCGAAAGGAATAGATGTAGATACGCTCAAACCACCTTC
TTGAAGTTGGAACCTC
>13c-3-1
GGGCGGTGTACCATACGACGTACCAGATTACGCTCATATGACAAGTTTGTACAAAAAAGTT
GGAGTGGATGGTTGTGATAGGAGGTTCAGTATGAAGGCAAATATGCAGCGGCATGTTAAG

GAAATTCATGAGGATGAAAATGCTAGTAAGAGCAACCAGCAGTTTATTTGTAAGGAGGAG
GGCTGCAACAAGGTGTTGAAGTATTCATCAAAGCTGAAGAAACATGAGGAATCCCATGTT
AAATTGGACTATGTGGAGGTATTGTGCGGTGAACCTGGCTGCATGAAGATGTTTACAAAC
GTTGAATATCTGAGGGTTCATAACCAATCTTGCCATCAGTATATTCAGTGTGAGATATGTGG
AGTAAAGCACCTGCTCTGGTTCGTCAAGTGCTCTTTGTTGTCCCTTGACTATGACTCCATT
GGCATACTCTTGGTTTTCTTTATTAACAGAAGCGTTTGCTCTATCTTTTTCTTTTGCTAAATG
ATCACGCCTAATAGTTGGGGTTTTTC
