# Supplementary material for: Functional characterization and analysis of transcriptional regulation of sugar transporter SWEET13c in sugarcane Saccharum spontaneum
Source: BMC Plant Biol. 2022 Jul 22;22:363. doi: 10.1186/s12870-022-03749-9 (PMC9308298; doi:10.1186/s12870-022-03749-9)
Supplement: Supplementary file 8 — Additional file 8. Pearson correlations between SsWEET13c and transcription factors expression pattern. [file 12870_2022_3749_MOESM8_ESM.pdf]

Additional file 8: Pearson correlations between *SsWEET13c* and transcription factors expression pattern

		leaf developmental gradient		diurnal cycles		different developmental stages	
Gene	Correlations TFs	Pearson Correlation	p value	Pearson Correlation	p value	Pearson Correlation	p value
SsWEET13c	*SsMYR2*	0.71	0	-0.28	0.25	0.00	1.00
	SsMADS34	0.42	0.12	0.54	0.02	-0.76	0.45
	SsWRKY18	0.74	0	0.4	0.09	-0.99	0.08
	SsHB36	-0.25	0.37	-0.03	0.91	-0.95	0.20
	SsABS5	-0.64	0.01	0.04	0.88	-0.82	0.39
	SsbZIP44	-0.41	0.13	-0.61	0.01	0.58	0.60
	SsHHO2	0.06	0.84	-0.06	0.79	0.27	0.83
	SsMYBS1	-0.19	0.5	-0.09	0.72	-0.99	0.07
	SsNID1	0.68	0.01	0.18	0.47	1.00	0.03
	SsRAP2.4	0.86	<0.0001	0.42	0.07	0.33	0.79
	SsbHLH035	0.89	<0.0001	0.08	0.73	-0.37	0.76
	SsDEL1	-0.27	0.33	-0.22	0.34	-0.92	0.26
	SsbHLH34	0.57	0.03	0.71	0	1.00	0.04
	SsERF4	-0.61	0.02	-0.42	0.07	-0.99	0.09
	SsILR3	0.88	<0.0001	0.75	0	-1.00	0.03
	SsKUA1	0.7	0	0.78	<0.0001	0.54	0.64
	SsTFIIIA-a	0.6	0.02	-0.07	0.79	1.00	0.01
	SsTFIIIA-b	-0.7	0	-0.49	0.03	0.96	0.19
	SsWRKY123	0.96	<0.0001	0.5	0.03	-0.95	0.21
	SsbZIP23	-0.85	<0.0001	0.55	0.01	-1.00	0.00

Note: In different developmental stages, the Pearson correlations between *SsWEET13c* and transcription factor were calculated in the stems.
