# Supplementary figures and images for: Functional characterization and analysis of transcriptional regulation of sugar transporter SWEET13c in sugarcane Saccharum spontaneum
Source: BMC Plant Biol. 2022 Jul 22;22:363. doi: 10.1186/s12870-022-03749-9 (PMC9308298; doi:10.1186/s12870-022-03749-9)

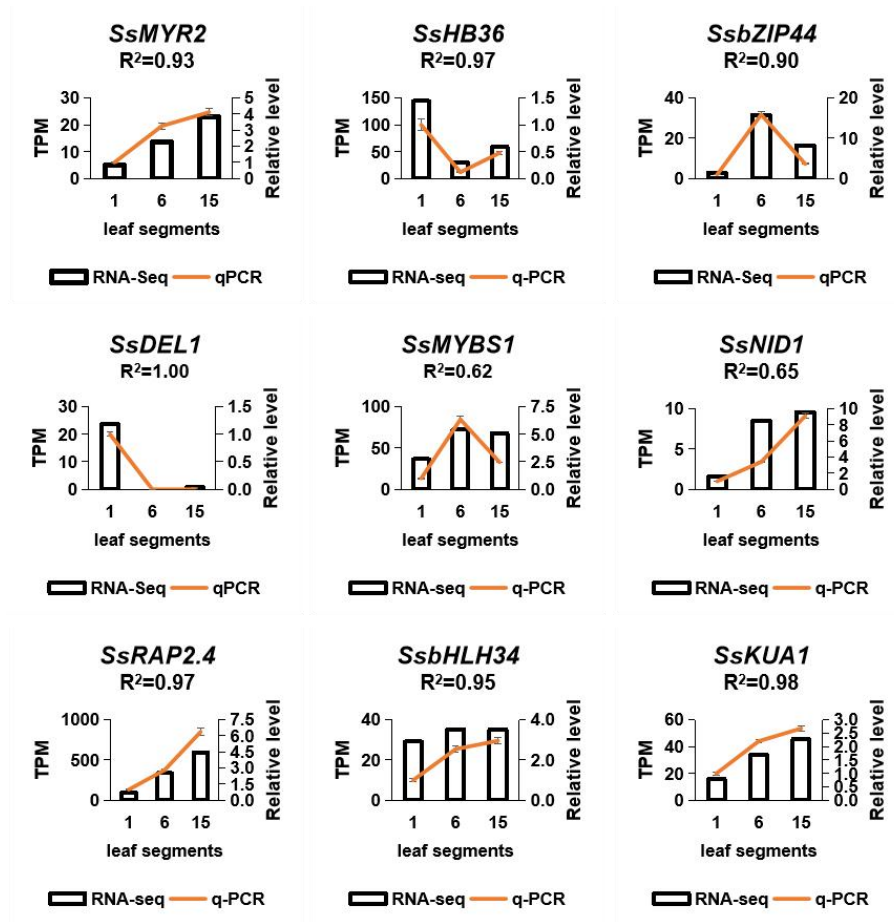

Additional file 9: RT-qPCR verification of nine TFs in partial segments of leaf gradients.

Supplement: Supplementary file 9 — Additional file 9. RT-qPCR verification of nine TFs in partial segments of leaf gradients. [file 12870_2022_3749_MOESM9_ESM.pdf]
