# Supplementary material for: Functional characterization and analysis of transcriptional regulation of sugar transporter SWEET13c in sugarcane Saccharum spontaneum
Source: BMC Plant Biol. 2022 Jul 22;22:363. doi: 10.1186/s12870-022-03749-9 (PMC9308298; doi:10.1186/s12870-022-03749-9)
Supplement: Supplementary file 10 — Additional file 10. Primers for amplifying the coding sequence of SsSWEET13. [file 12870_2022_3749_MOESM10_ESM.pdf]

Additional file 10: Primers for amplifying the coding sequence of *SsSWEET13*.

Gene name	Upstream primer	Downstream primer
SsSWEET13c	ATGGCAGGCCTATCTCTGCAGC	CTAGACCACATGGACGGCGG
