# Supplementary material for: Functional characterization and analysis of transcriptional regulation of sugar transporter SWEET13c in sugarcane Saccharum spontaneum
Source: BMC Plant Biol. 2022 Jul 22;22:363. doi: 10.1186/s12870-022-03749-9 (PMC9308298; doi:10.1186/s12870-022-03749-9)
Supplement: Supplementary file 11 — Additional file 11. The primers for RT-qPCR of SsSWEET13c and nine TFs from yeast one-hybrid in S. spontaneum. [file 12870_2022_3749_MOESM11_ESM.pdf]

Additional file 11: The primers for RT-qPCR of *SsSWEET13c* and nine TFs from yeast one-hybrid in *S. spontaneum*.

Gene name	Upstream primer	Downstream primer
SsSWEET13c	TGGCTATGAGGACAAGACT	AAGATTGATGGGAGCAGAT
SsMYR2	AGGGTCAGTCGGTGTAGAA	CAATCTCCTCAAAGCCTGTAAATG
SsHB36	TACAGGGTGGTACTCTTCTTGA	GAAGCTCCCGTGGAAGAATAC
SsbZIP44	GTCTAGGCTAAGGAAGCAACAA	ACAAGGTTCTGGCTGGTTATG
SsDEL1	CAGAGTGGTGGTGATGTCTTG	CAGGGTGGAAAGGACCATAAA
SsMYBS1	GAAGAGGAGCACAGGTTGTT	GTCCGTGAGATGACGAAGTT
SsNID1	CGTTGGTCCCTGTTCCATTA	CTGCCTTAATCACCTCCTTACC
SsRAP2.4	GTACTTCAACGCACACCAGA	ATGGATGGAGAGGACGAGTAG
SsbHLH34	CGTATGGTGGCACAACTTAGA	GCTCATTCTTCTCCTCCTTCAG
SsKUA1	GACATCTCAGCCTATGGAACAG	TGGTAGTATGGAGGAGGAATCA
